# Random Mutagenesis MAPPIT Analysis Identifies Binding Sites for Vif and Gag in Both Cytidine Deaminase Domains of Apobec3G

**DOI:** 10.1371/journal.pone.0044143

**Published:** 2012-09-10

**Authors:** Isabel Uyttendaele, Delphine Lavens, Dominiek Catteeuw, Irma Lemmens, Celia Bovijn, Jan Tavernier, Frank Peelman

**Affiliations:** 1 Department of Medical Protein Research, VIB, Ghent, Belgium; 2 Department of Biochemistry, Faculty of Medicine and Health Sciences, Ghent University, Ghent, Belgium; King's College, London, United Kingdom

## Abstract

The mammalian two-hybrid system MAPPIT allows the detection of protein-protein interactions in intact human cells. We developed a random mutagenesis screening strategy based on MAPPIT to detect mutations that disrupt the interaction of one protein with multiple protein interactors simultanously. The strategy was used to detect residues of the human cytidine deaminase Apobec3G that are important for its homodimerization and its interaction with the HIV-1 Gag and Vif proteins. The strategy is able to identify the previously described head-to-head homodimerization interface in the N-terminal domain of Apobec3G. Our analysis further detects two new potential interaction surfaces in the N-and C-terminal domain of Apobec3G for interaction with Vif and Gag or for Apobec3G dimerization.

## Introduction

To reveal how proteins interact in a protein complex, the detailed structure of the complex is ideally determined via crystallography methods or NMR. However, the structure determination of protein complexes remains challenging and the number of complex structures lags far behind the number of known protein interactions [1]. This gap will grow as interactomics projects lead to a vast increase in the number of known protein-protein interactions. Alternative methods are developed for prediction of protein complex structures to -at least partially- bridge this gap. In silico methods such as homology based modeling and protein-protein docking can predict the structure of protein complexes [1]–[4]. Additionally, fitting of monomer structures or models into low resolution structures of the complex obtained via SAXS, cryo-electron microscopy or electron tomography can provide a model for the complex [5]–[7]. Models from these predictions can further be validated by experimental methods, such as mutagenesis of the predicted interface(s) combined with a method to detect the specific protein-protein interaction. Conversely, experimental identification of interface residues can help to guide the docking process in data-driven docking, often resulting in better models [8]. The development of new methods to determine interfaces in protein-protein interactions can thus contribute to the development of alternative methods for complex structure modeling. We here propose a new random mutagenesis strategy to identify putative interface residues based on the mammalian two-hybrid method MAPPIT.

MAPPIT is a two-hybrid method based on reconstitution of cytokine receptor signaling for the detection of protein-protein interactions [9]. The MAPPIT principle is outlined in figure S1 in supporting information. We previously used MAPPIT and site directed mutagenesis to identify an interface in the human host restriction factor Apobec3G that is important for its dimerization and its interaction with the HIV-1 protein Vif [10]. Human apolipoprotein B messenger RNA-editing catalytic polypeptide-like G (Apobec3G) is a member of the Apobec protein family of cytidine deaminases [11]. Apobec3G is a host restriction factor that inhibits the infectivity of HIV-1 virus particles that lack the accessory protein virion infectivity factor (Vif) [12]. Apobec3G is incorporated into newly formed HIV-1 virions and catalyzes cytidine deamination during reverse transcription of the viral genome in infected cells. This leads to hypermutation and degradation of the newly synthesized viral DNA [13]–[16]. Apobec3G further restricts HIV-1 infection through deaminase-independent mechanisms [17]–[26]. Unfortunately, HIV-1 can efficiently counteract the restrictive effects of Apobec3G by Vif. HIV-1 Vif is a 23 kDa protein that targets Apobec3G for proteasomal degradation [27]–[31]. Vif binds to Apobec3G and recruits via its SOCS box domain an E3 ubiquitin ligase complex with Cullin-5, Elongin B, Elongin C and Rbx1 subunits [32], [33]. This leads to the ubiquitination of Apobec3G and degradation by the 26S proteasome.

Apobec3G contains two characteristic cytidine deaminase (CDA) domains [34]. Only the C-terminal CDA domain (CD2) is catalytically active in cytidine deamination, whereas the N-terminal CDA domain (CD1) is involved in nucleic acid binding and virion incorporation [19], . Virion incorporation of Apobec3G is mediated via the RNA-dependent interaction with the conserved nucleocapsid domain of the HIV-1 Gag protein. The nucleocapsid domain is necessary and sufficient for interaction with and incorporation of Apobec3G in virus-like particles [38]–[43]. The structure of the CD2 domain of Apobec3G has been determined by X-ray crystallography and NMR [44] [48]. This Apobec3G domain folds into a five-stranded β sheet flanked by six α helices. Several homology models have been proposed for the CD1 domain [10], . In the crystal structure of the related Apobec2, its single deaminase domain forms tetramers via two types of interactions: two domains interact symmetrically by pairing of their β2 strands. Two dimers further form tetramers via a symmetrical head-to-head interface containing residues of the α1-β1 and β4-α4 loops and the α6 helix [53]. A similar head-to-head interface was proposed and identified for the N-terminal domain of Apobec3G [10], [51], [52]. Mutations in this interface affect multiple aspects of Apobec3G function including dimerization, virion incorporation, cellular localization and interaction with Vif [10], [51], [52]. Using MAPPIT, homology modeling and site directed mutagenesis we mapped residues in this dimerization interface in CD1 of Apobec3G that are important for the Apobec3G-Apobec3G interactions [10].

Here, we tested the effect of mutations in the dimerization interface on the interaction between Apobec3G and Gag. We present a new strategy to screen for mutations that disrupt a protein-protein interaction based on random mutagenesis in combination with MAPPIT. The strategy allows evaluating the effect of many random mutations in one protein on interaction with multiple interaction partners in parallel. Using this strategy, we identified regions in both Apobec3G domains that are involved in the interaction of Apobec3G with Apobec3G, Vif and Gag.

## Results

### MAPPIT-analysis of the Apobec3G-Gag interaction reveals a similar interaction pattern as Apobec3G homomerization

We previously used MAPPIT to demonstrate the importance of the head-to-head interface of the Apobec3G N-terminal domain for the Apobec3G-Apobec3G and the Apobec3G-Vif interaction [10]. Here, we used MAPPIT to study the role of this interface in the Apobec3G-Gag interaction.

**Figure 1:**
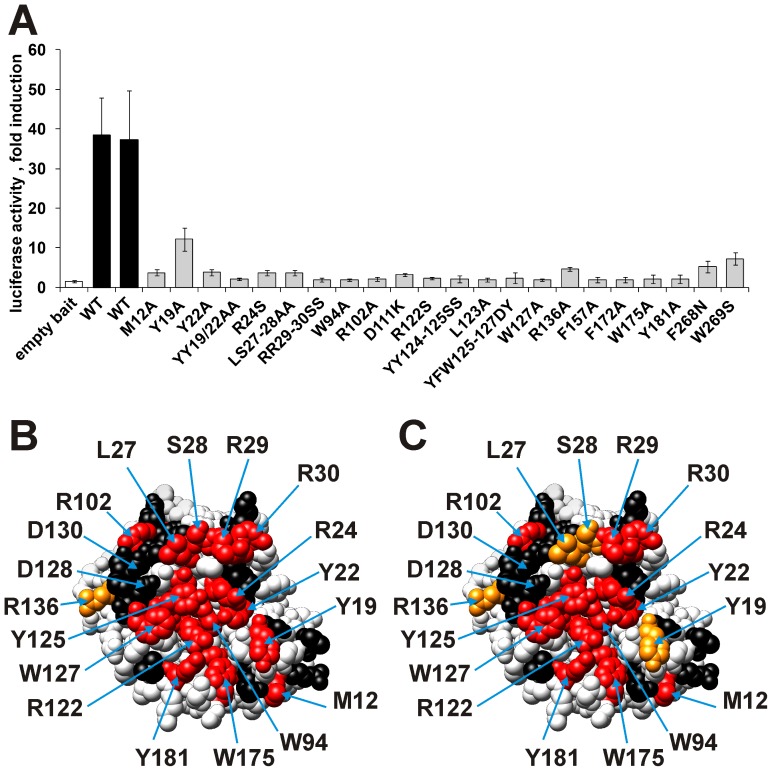
Effect of site-directed mutations in the N-terminal CDA domain of Apobec3G on the interactions with Gagpol and Gag. A: Effect of mutations on the Apobec3G-Gag interaction. Interactions between the Gag prey and the different mutant Apobec3G baits were determined via MAPPIT. The data are expressed as fold induction of luciferase activity after stimulation with Epo. All mutations that disrupt the Apobec3G-Gagpol interaction (Red/Orange in panel C) also disrupt the Apobec3G-Gag interaction. B &C: The effects on the Apobec3G-Apobec3G (B) and Apobec3G-Gagpol (C) interaction were determined via MAPPIT. The residues of the head-to-head interface are directed towards the viewer. B: Effect of mutations on the Apobec3G-Apobec3G interaction (47). C: Effect of mutations on the Apobec3G-Gagpol interaction. The colors in A and B indicate the relative MAPPIT signal of the Apobec3G bait mutants, compared to wild type. Color codes: Red: <25% of WT, orange: <50% of WT, black: >50% of WT (no strong effect). Mutations that disrupt the Apobec3G-Apobec3G interaction (Red/Orange) also disrupt the Apobec3G-Gagpol interaction.

**Figure 2:**
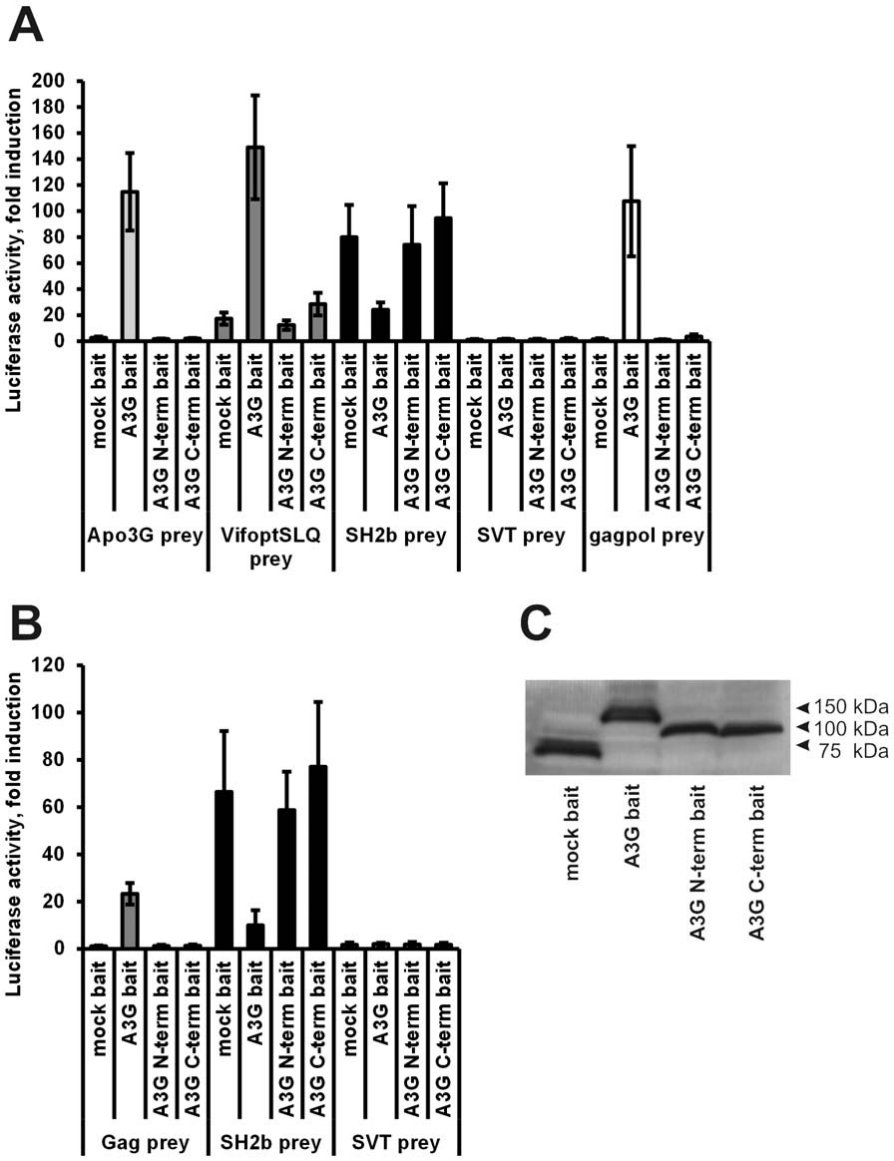
Importance of the N- and C-terminal domain of Apobec3G for the interaction with Apobec3G, Vif, Gag and Gagpol determined. A&B: Apobec3G, its N-terminal domain or its C-terminal domain were coupled to a V5-tagged MAPPIT bait receptor. Via MAPPIT, their interactions were determined with preys for full-length Apobec3G, Vif, Gagpol and Gag. The data are expressed as fold induction of luciferase activity after stimulation with Epo. C: Expression of the baits in panel A and B was determined via Western blot using an anti-V5 tag antibody. All baits are properly expressed.

MAPPIT allowed the detection of the interaction of the Apobec3G bait with Gagpol and Gag, which originates from the Gagpol precursor polyprotein, coupled as prey. The Gagpol prey led to a robust MAPPIT signal, while the Gag prey resulted in a modest but reproducible MAPPIT signal (Figure 1A and 2). A MAPPIT prey with a truncated Gag (amino acids 1−377) misses the Gag nucleocapsid domain and showed no MAPPIT signal (figure S2). This is in line with the important role of the nucleocapsid domain in the Apobec3G-Gag interaction [38]–[43]. In our previous study, a panel of Apobec3G bait mutants in the head-to-head interface of the N-terminal domain was generated. We tested the association of these Apobec3G mutants with the Gagpol prey and confirmed the data with the Gag prey. The effect of Apobec3G bait mutations on the interaction with Gagpol or Gag is quasi identical to their effect on the interaction with Apobec3G (Table 1 and Figure 1B and 1C) [10]. This observation indicates that mutations that disrupt the head-to-head interaction of Apobec3G N-terminal domains also disrupt the interaction with Gag. The RNA-mediated head-to-head interaction of two Apobec3Gs may be required for Gag binding.

**Table 1:** Overview of the effect of mutations in the Apobec3G MAPPIT bait on the interaction with the Gagpol prey.

	Gagpol %WT	stdev
**V9A**	101	14
**R11S**	83	17
**M12A**	25*	13
**R14S**	106	46
**Y19A**	36*	18
**YNFY19-22ANFA**	2*	2
**N20A**	71	16
**Y22A**	15*	4
**R24S**	19*	10
**LS27-28AA**	48*	20
**RR29-30SS**	2*	1
**N31S**	78	8
**T32Q**	118	44
**T32D**	111	31
**T32E**	81	15
**W34A**	50	15
**R55A**	64	28
**K63E**	104	51
**W94A**	4*	3
**TK98-99AS**	79	20
**TK98-99AD**	74	12
**K99D**	96	24
**R102A**	1*	1
**RD102-103SS**	63	16
**RD102-103EK**	85	50
**TF106-107AA**	94	20
**E110S**	80	14
**E110K**	84	25
**D111K**	9*	8
**R122S**	1*	0
**L123A**	2*	1
**Y124A**	61	20
**YY124-125SS**	1*	1
**YFW125-127DY**	1*	1
**W127A**	1*	1
**D128K**	98	23
**D128Q**	71	8
**D128H**	60	16
**P129A**	70	23
**D130A**	103	58
**QE132-133AA**	80	25
**R136A**	48*	29
**S137A**	127	37
**F157A**	4*	3
**YS166-167AA**	80	19
**F172A**	3*	2
**W175A**	2*	1
**Y181A**	3*	1
**L184A**	110	21

MAPPIT luciferase fold inductions for each mutant are expressed as percentage of wild type Apobec3G bait. The averages of several independent MAPPIT experiments and standard deviation (SD) are shown. Strongly decreased values that are significantly different from the WT in a paired t-test are indicated by an asterisk. HEK293T cells were transiently co-transfected with plasmids encoding the chimeric Apobec3G WT or mutant Apobec3G bait constructs and Gagpol prey constructs, combined with the pXP2d2-rPAP1-luci reporter. The transfected cells were either stimulated for 24 h with Epo or were left untreated. Luciferase measurements were performed in triplicate. Data are expressed as a percentage of the WT fold inductions (Epo-stimulated/unstimulated).

We showed before that four mutations at the edge of the head-to-head interface specifically affected the Apobec3G-Vif interaction: D128K and P129A disturbed the interaction with Vif whereas a T32Q and a K99D mutation increased the MAPPIT signal [10]. These mutations do not affect the Apobec3G-Gag interaction, which shows that the mutated residues are specifically involved in the interaction with Vif.

### Both N- and C-terminal domains of Apobec3G are involved in the interactions of Apobec3G with Apobec3G, Vif and Gag

To investigate the role of the N- and C-terminal domains of Apobec3G in the interaction with Apobec3G, Vif and Gag, the N- and C-terminal domains of Apobec3G were coupled as MAPPIT baits. The interaction of these baits with four different MAPPIT preys containing respectively codon optimized Vif SLQ144-146AAA, wild type Apobec3G, Gagpol and SH2-Bβ, was tested. The SLQ144-146AAA mutation in the Vif prey prevents proteasomal degradation of the Apobec3G bait, and results in a higher MAPPIT signal [32]. We will further refer to this prey as the ‘Vif prey’. The SH2-Bβ prey is used as a positive control to test the expression of the bait mutants. SH2-Bβ interacts with JAK2 bound to the bait receptor [54] and the SH2-Bβ prey therefore gives a positive MAPPIT signal with any MAPPIT bait that is expressed at the plasma membrane. The pMG2-SVT prey is used as a negative control in our experiments.

While most reports show the interaction of Vif with the Apobec3G CD1 domain [37], [55], [56], our assay demonstrates that Vif may also be able to interact with the C-terminal domain of Apobec3G (Figure 2A). However, the MAPPIT signal is much more robust with the full-length Apobec3G bait. The isolated N- or C-terminal Apobec3G domains do not interact with Gag (Figure 2B). We could not show interaction between the full-length Apobec3G prey and the isolated N-terminal or C-terminal domain of Apobec3G as bait (Figure 2A). This suggests that the head-to-head interaction between the N-terminal domains is promoted by the presence of the C-terminal domain. Based on these data, we propose that the N- and C-terminal domains both play a role in Apobec3G dimerization or oligomerization and in the interaction with Vif and Gag. We therefore further identified the exact binding sites in both domains as described below.

### Development of a random mutagenesis MAPPIT strategy and its application for analysis of the Apobec3G –Apobec3G, Apobec3G-Vif and Apobec3G-Gag interactions

**Figure 3:**
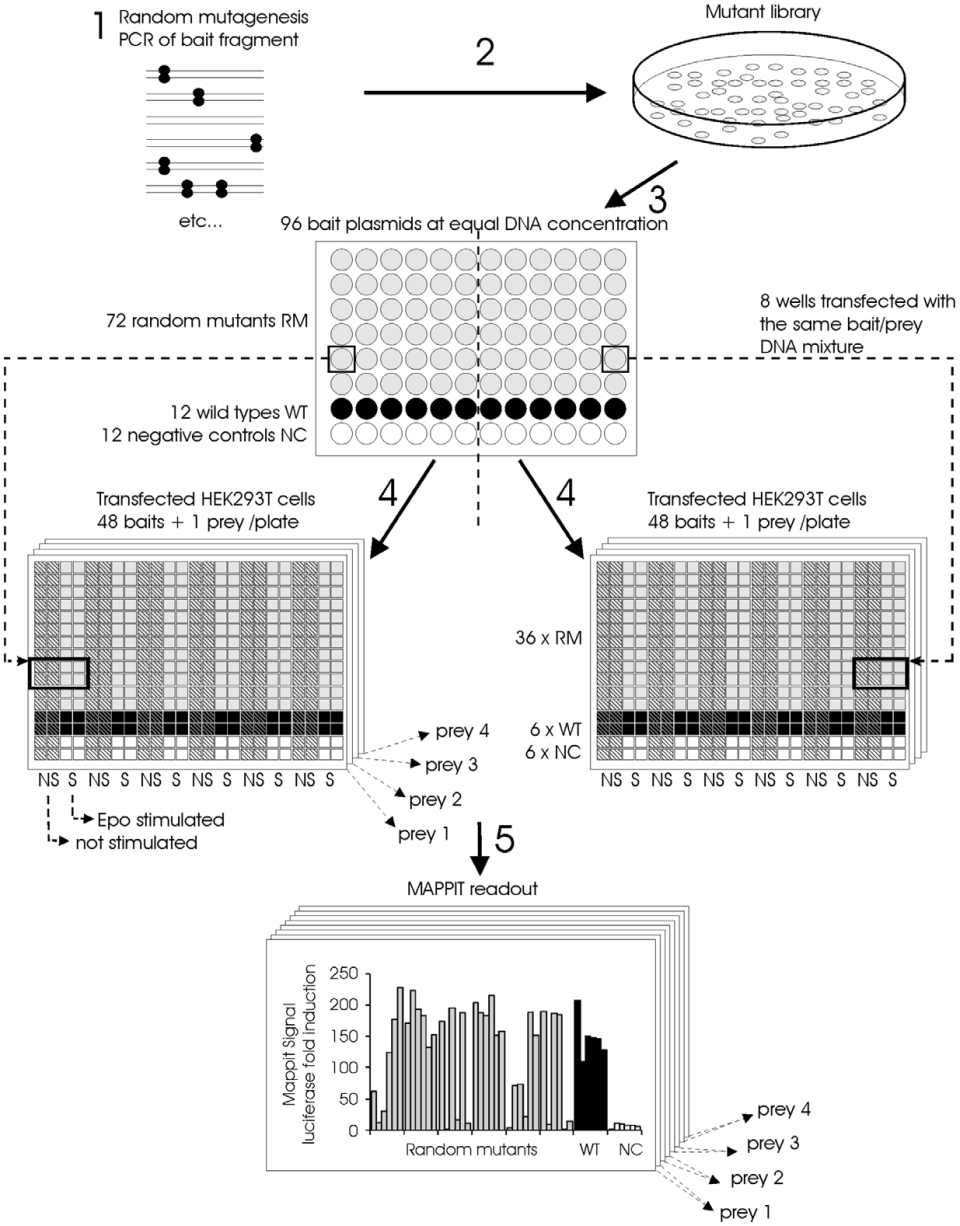
Method for the identification of random mutants that disrupt a protein-protein interaction based on MAPPIT. 1: A fragment of the MAPPIT bait is randomly mutated via Genemorph® II PCR. 2: The mutated PCR fragments are ligated into the MAPPIT bait vector and the resulting plasmid mutant pool is used to transform E. coli. 3: Plasmid DNA from the resulting colonies is prepared via automated 96-well DNA miniprep. Each 96-well miniprep plate contains DNA from colonies of 72 mutants, 12 wild types and 12 negative controls. The DNA concentration in all DNA samples is normalized to the same concentration. The resulting DNA is used to transfect HEK293T cells via an automated procedure using liquid handling robots. 4: Each bait is co-transfected separately with four different MAPPIT preys and the luciferase reporter. Each bait/prey mixture is used to transfect 8 wells of a 384-well plate with Hek293T cells. One 96-well MAPPIT bait miniprep plate in combination with four MAPPIT preys thus leads to 8 transfected 384 well plates. Each transfected 384 well plate contains a single MAPPIT prey in combination with 6 wild type baits, 6 negative control baits and 36 random bait mutants. 5: From each transfected bait/prey mixture, four wells are stimulated with Epo, the remaining four wells are not stimulated. After overnight Epo stimulation, the luciferase activity is determined via a luminescence reader. The MAPPIT signal is calculated by dividing the signal of the four stimulated wells by the signal of the four unstimulated wells.

Our mapping of binding sites in Apobec3G via mutagenesis was hitherto restricted to the predicted head-to-head interface in the N-terminal domain of Apobec3G. To extend this mapping to the entire Apobec3G protein, we developed a method to screen for mutations that disrupt protein-protein interactions via a combination of random mutagenesis and MAPPIT. In this method, we randomly mutate the MAPPIT bait insert via error prone PCR and test the interaction of the different mutants with several preys by co-transfection of HEK293T cells in 384-well format (Figure 3).

To screen for mutations in Apobec3G that decrease the interaction with Apobec3G, Vif or Gag, two slightly overlapping fragments of Apobec3G (amino acids 1 to 178 and amino acids 175 to 384) were mutated separately. 1152 potential bait mutants from each mutagenesis were isolated. The interaction of these 2304 potential bait mutants with four different MAPPIT preys containing respectively codon optimized Vif SLQ144-146AAA, wild type Apobec3G, Gagpol and SH2-Bβ, was tested.

**Figure 4:**
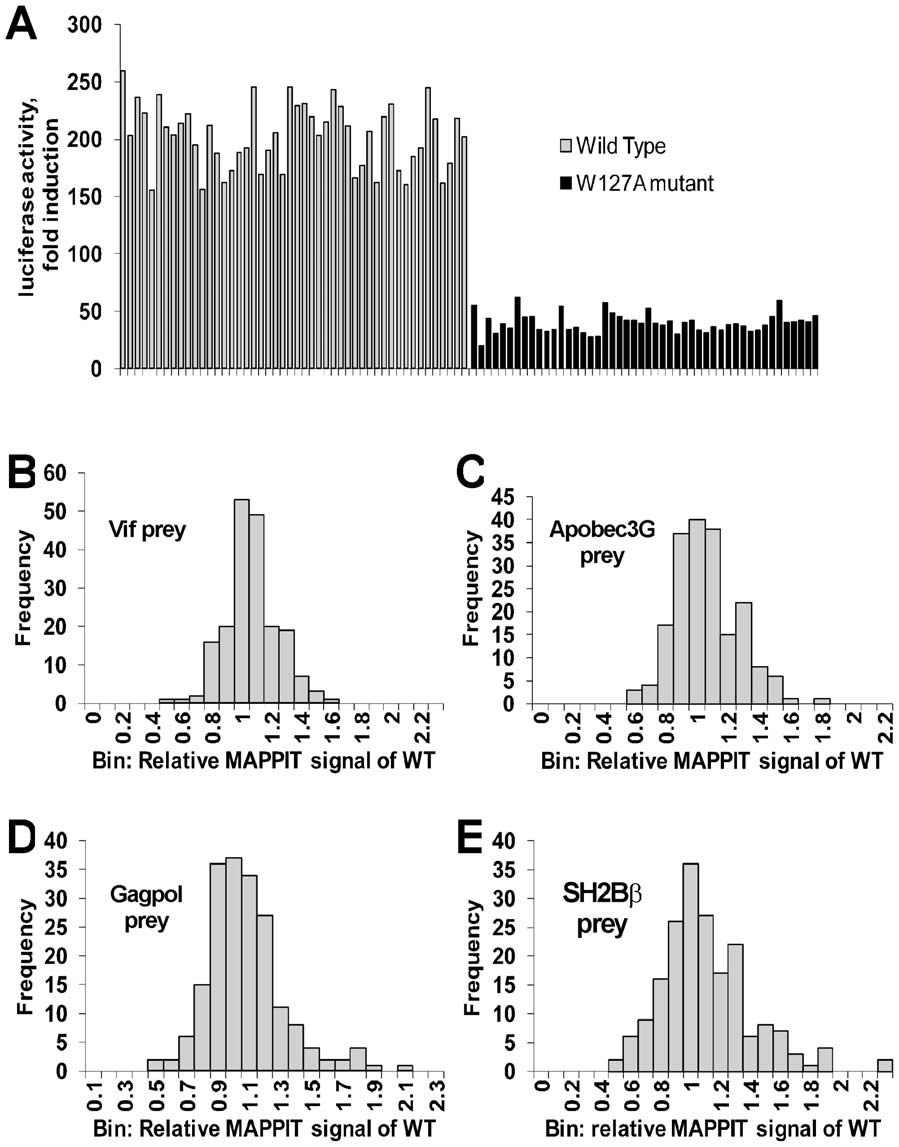
Proof of principle and variation of wild type in the random mutagenesis experiments. A: As proof of principle, 48 different colonies of the WT and W127A Apobec3G bait were picked and their DNA was miniprepped. These constructs were transiently transfected in Hek293T cells in 384-well plates in combination with plasmids encoding the Vif prey and the pXP2d2-rPAP1-luciferase reporter. The transfected cells were either stimulated for 24 h with Epo or left untreated. Luciferase measurements were performed in quadruplicate. Data are presented as fold inductions of luciferase measurements. The method allows a clear discrimination between all WT and the W127A mutants. B,C,D,E: histograms of the relative MAPPIT signal of all wild type Apobec3G baits with the four preys in our random mutagenesis experiments. The relative MAPPIT signal of each wild type was calculated as described in the materials and methods section. The histograms of the relative MAPPIT signal of the wild types gives an estimate of the variation of the WT signal in our mutant screens. The relative MAPPIT signal is always higher than 0.45.

In a proof of principle experiment, the capability of the strategy to distinguish between the interaction of the Vif prey with the wild type and the W127A mutated Apobec3G MAPPIT bait, was examined. As shown in figure 4A, the method clearly allows discriminating between the wild type and the mutant colonies.

In the random mutagenesis MAPPIT screen, each transfection was performed in triplicate and the relative MAPPIT signal of the mutants compared to six control wild types was calculated as described in the materials and methods section. To estimate the variation of the relative MAPPIT signal for wild type Apobec3G, every wild type control was compared first with the median of the five other wild type controls on the plate. In this analysis the relative MAPPIT signal of every wild type is higher than 0.45 (Figure 4B, C, D, E). All mutants with a relative MAPPIT signal ≤0.65 for the Vif, Apobec3G or Gagpol preys plus>0.5 for the SH2-Bβ prey were sequenced. The resulting single amino acid substitutions are listed in tables S2 and S3 in supporting information. A relative MAPPIT signal below 0.45 strongly suggests that the mutant affects the interaction, a relative MAPPIT signal between 0.45 and 0.65 may indicate a weaker effect on the interaction. The mutations and their relative MAPPIT signal with the three interactions partners were mapped on a homology model for the N-terminal domain and on the crystal structure of the C-terminal domain. Surface areas where mutations with low relative MAPPIT signals cluster, indicate possible interaction surfaces. Selected mutations in these areas were retested in additional MAPPIT assays to confirm the effect of the mutation.

Surprisingly, the relative MAPPIT signal of the SH2-Bβ prey for wild type Apobec3G is lower than for many of the Apobec3G bait mutants including the W127A mutant. Although this suggests a lower expression level for wild type Apobec3G, Western blot analysis confirms the proper expression of the wild type and mutant Apobec3G baits (figure S3). The luciferase values after Epo stimulation are very similar for wild type and mutant baits with the SH2-Bβ prey. (figure S4). The lower fold induction for the wild type bait is rather caused by an unusually high luciferase reporter activity of the unstimulated wild type bait. This may mean that homodimerization of Apobec3G in the wild type bait is able to partially activate JAK2 of the bait receptor from the cytosolic side, leading to luciferase reporter activity with the SH2-Bβ prey in the absence of Epo ligand. Bait mutations that affect the Apobec3G homodimerization do not show this effect.

### Random mutagenesis of the N-terminal CDA domain of Apobec3G

**Figure 5:**
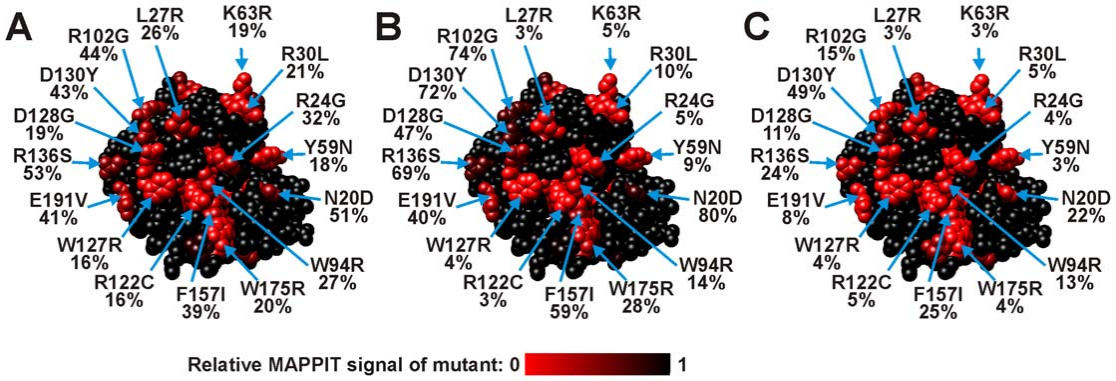
Position and effect of random mutations in the Apobec3G N-terminal CDA domain bait on MAPPIT interaction with a Vif, Apobec3G or Gagpol prey. The residues of the head-to-head interface are directed towards the viewer. The effect of the mutation is indicated via a color scale from black to red, as indicated below the models. Color codes: black: no mutation at this position or 100% of WT (no effect of the mutation), Red: 0% of WT. The relative MAPPIT signal (expressed as % of Wild Type) is shown below the mutation indicator. A: Effect of mutations on the Apobec3G-Vif interaction. B: Effect of mutations on the Apobec3G-Apobec3G interaction. C: Effect of mutations on the Apobec3G-Gagpol interaction.

In the N-terminal domain, 81 different single amino acid substitutions were identified that affect the MAPPIT signal with one or more of the preys (Table S2 in supporting information). The 81 mutations correspond to 58 residues in the N-terminal domain. All these mutations disturbed the interaction with both Vif, Apobec3G and Gagpol, in line with our previous observation from the mutagenesis analysis of the head-to-head interface. None of the single amino acid substitutions had a specific effect on one of the three interactions. Only a combined A51V/P129S mutation specifically affected the Apobec3G-Vif interaction, which is probably due to the mutation of residue P129. Figure 5 shows a map of the mutations on a model for the Apobec3G N-terminal domain. Half of the mutations occur at 28 buried residues and probably influence the structure, folding or stability of the N-terminal domain. 30 mutated residues are surface exposed (>20% RSA).

The surface exposed residues that strongly affected the three interactions were all located in the predicted head-to-head interface for Apobec3G homodimerization and corresponded with those obtained previously in the site-directed mutagenesis study of the Apobec3G-Vif, Apobec3G-Apobec3G [10] and Apobec3G-Gag interactions (Table 1, Figure 1, Figure 5). This shows that the random mutagenesis MAPPIT approach is very well able to detect the interface areas of this protein-protein interaction.

**Figure 6:**
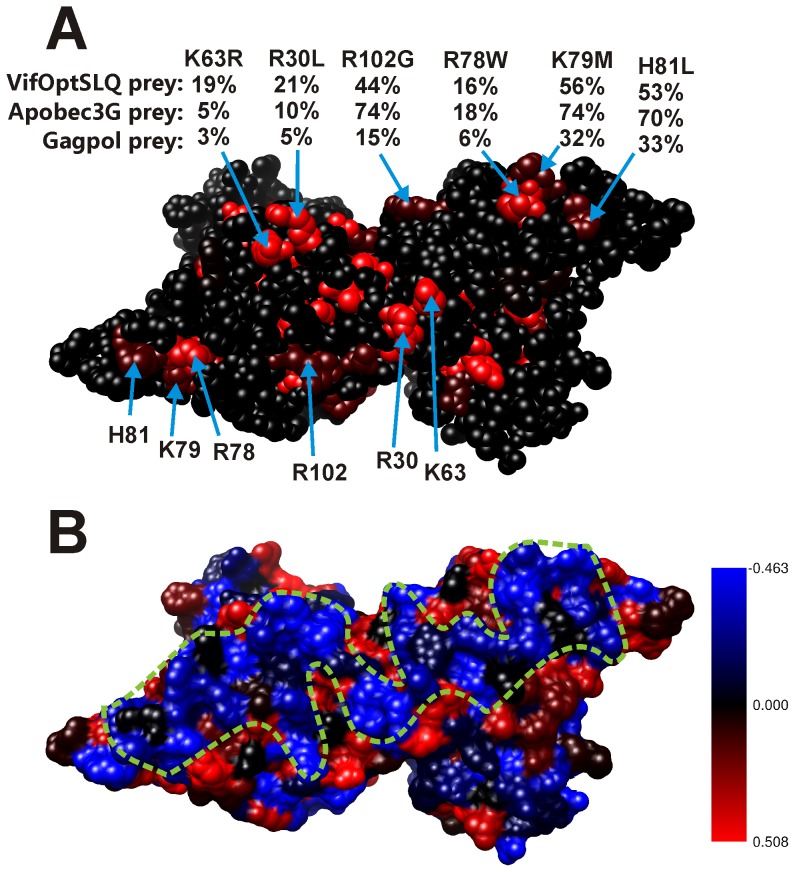
Position and effect of random mutations in the Apobec3G N-terminal CDA domain, modeled as a head-to-head dimer. A: The effect of mutations on the Apobec3G-Apobec3G interaction is indicated via a color scale from black to red, as in figure 5. Color codes: black: no mutation at this position or 100% of WT (no effect of the mutation), red: 0% of wild type. For each mutation, the relative MAPPIT signals (expressed as % of Wild Type) for interaction with the three preys are shown below the mutation indicator. From top to bottom, the numbers indicate the relative MAPPIT signal for interaction with the Vif prey, Apobec3G prey and the Gagpol prey. Mutations at the surface of the Apobec3G N-terminal CDA domain containing α helices 2, 3 and 4 are shown. In the dimer model, these regions are juxtaposed. B: These mutations coincide with a region with a high RNA binding propensity. The RNA binding propensity is shown by color code: blue: high RNA binding propensity, red: low RNA binding propensity. The putative RNA binding surface is indicated by a dashed green line.

Several mutations in Apobec3G that weakly affected the three interactions cluster in a region at the surface of the Apobec3G N-terminal domain containing α helices 2, 3 and 4. In a dimer model for the Apobec3G N-terminal domain, these regions are juxtaposed, and the mutations coincide with a region with a high RNA binding propensity (Figure 6). This second region may therefore form the RNA binding site of Apobec3G.

As the crystal structure of Apobec2 revealed that two Apobec2 molecules dimerize via pairing of the β2 strands, the β2 strand of the Apobec3G N-terminal domain was predicted to interact with the β2 strand of the C-terminal domain [51]. The lack of disruptive mutations in this area of the Apobec3G N-terminal domain suggests that the described interaction is not important for the tested interactions of Apobec3G.

### Random mutagenesis of the C-terminal CDA domain of Apobec3G

**Figure 7:**
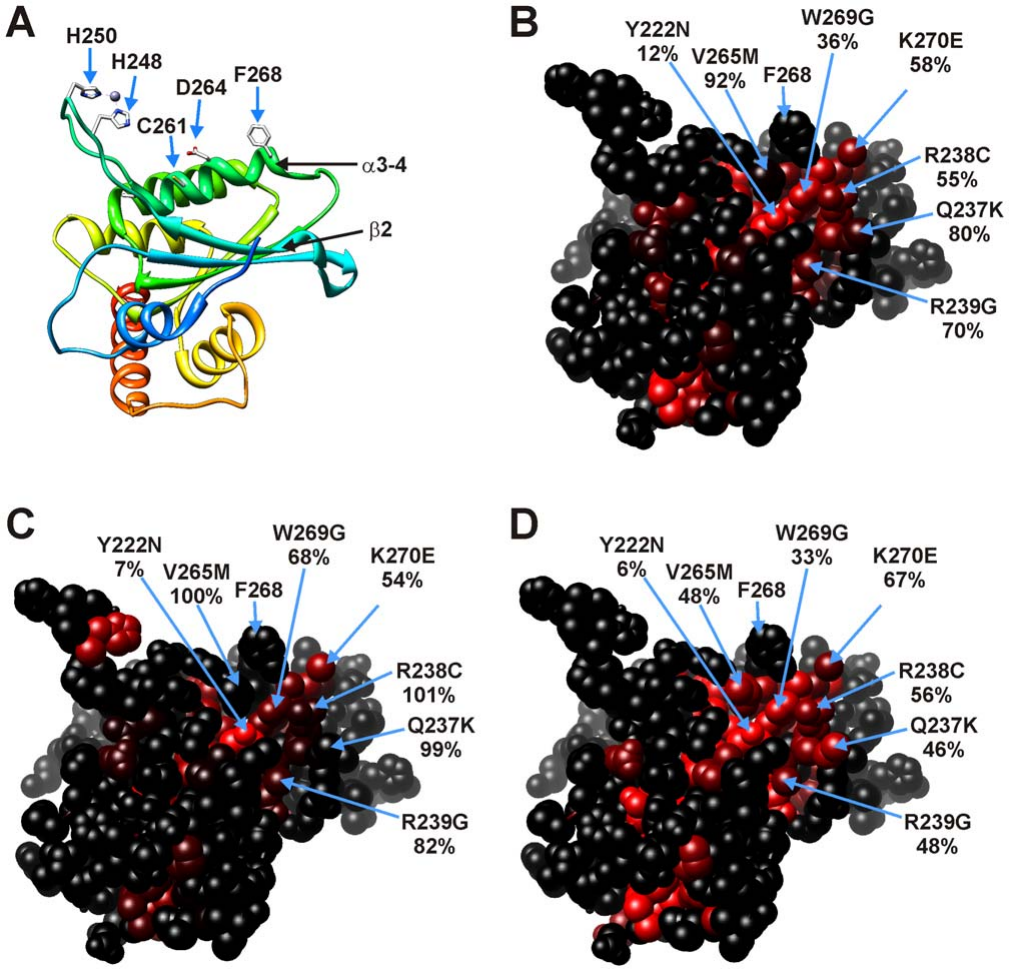
Position and effect of random mutations in the Apobec3G C-terminal CDA domain bait on MAPPIT interaction with a Vif, Apobec3G or Gagpol prey. The residues of the α2 and β2 region are directed towards the viewer. A: Ribbon model in the same orientation as in panels B−D. B: Effect of mutations on the Apobec3G-Vif interaction. C: Effect of mutations on the Apobec3G-Apobec3G interaction. D: Effect of mutations on the Apobec3G-Gagpol interaction. As in figure 5 and 6, the effect of the mutations in panel B−C is indicated via a color scale from black to red. Color codes: black: no mutation at this position or 100% of WT (no effect of the mutation), red: 0% of WT. The relative MAPPIT signal (expressed as % of Wild Type) is shown below the mutation indicator.

68 different single amino acid substitutions in the C-terminal domain of Apobec3G affected at least one of the tested interactions and corresponded to 56 different residues in the C-terminal domain (Table S3 in supporting information). 27 mutated residues were in the core of the protein (<20% RSA) and 29 are surface residues (>20% RSA). The effect of most mutations on the three interactions strongly coincided (Figure 7), as for mutations in the N-terminal domain. No disruptive mutations were found at the surface exposed residues of the α1-β1 and β4-α4 loops and the α6 helix of the C-terminal domain, indicating that a tail-to-tail type interaction of the C-terminal domains does not play a role in the Apobec3G interactions detected by our MAPPIT assays. In contrast, random mutations of Y222, Q237, R238, R239, G240, F241, V265, W269, K270 and a site-directed F268N mutation in the center of the β2 strand and the α2 helix affected the interaction with the Gagpol prey (Figure 7D and table S3 in supporting information). We showed a similar disruptive effect of F268N and W269S mutations on the interaction with the Gag prey (Figure 1A), confirming that this zone around the β2 strand and the α2 helix plays a role in the interaction with Gag. Mutations in this region also slightly affected the interaction with the Vif prey, suggesting that this region is also involved in the interaction with Vif (Figure 7B).

We tested the effect of combined mutations in the α2-β2 area of the Apobec3G bait on the interactions with the Vif, Gag, Gagpol and Apobec3G baits (Figure S5 in supporting information). The combined mutations drastically affect all interactions, including the interaction with the Apobec3G prey. This suggests that the α2-β2 area may also play a role in the Apobec3G-Apobec3G interaction. All these bait mutants seem to be properly expressed, as tested via the SH2-Bβ prey. The MAPPIT signal for the mutant baits with the SH2-Bβ prey is much higher than for the wild type bait, in line with our observations for mutations that disrupt the Apobec3G-Apobec3G interaction.

We confirmed the inhibitory effect of a F268N+K270E mutation on the Apobec3G-Vif and Apobec3G-Gag interaction in co-immunoprecipitation experiments (figure S6). However, the F268N+K270E mutant can be degraded by Vif like wild type Apobec3G when the mutant Apobec3G and Vif are co-expressed (data not shown).

F268 is found close to D264 and C261 in the α2 helix. D264 and C261 form an intermolecular Zn^2+^ bridge with H248 and H250 of another Apobec3G C-terminal domain in the crystal structure. A role for this zinc mediated intermolecular interaction in oligomerization of Apobec3G was proposed [46]. We therefore tested the effects of mutations of C261 and H248 on the interaction of the Apobec3G bait with the Gag and Gagpol preys (Figure S5 in supporting information). While the C261A mutation had a slight effect, the H248A mutation had no significant effect on the tested interactions, indicating that the Zn^2+^ bridge is probably not involved in the interaction with Gag and Gagpol.

## Discussion

By combining error prone PCR, automated 96-well DNA minipreparation, automated 384-well transfections and MAPPIT, we developed a strategy to screen for random mutations that disrupt protein-protein interactions in intact living cells. Using this strategy, 149 single amino acid substitutions that affected interactions of Apobec3G were identified. The random mutagenesis MAPPIT approach allowed the detection of the previously identified head-to-head interface in the CD1 domain as the most prominent zone for Apobec3G-Apobec3G interaction. This demonstrates that our random mutagenesis approach is able to detect putative interaction interfaces in a way that is not biased by a priori assumptions or models. Besides the known head-to-head interface, the study suggests a new interface that may be involved in RNA-mediated dimerization in the N-terminal domain. Moreover, we defined a putative interface for Gag and Vif binding in the C-terminal domain.

In the random mutagenesis MAPPIT approach, the interaction surfaces of a bait protein with 4 different interacting preys can be analyzed in parallel in the transfection experiment. After determining the best conditions for error prone PCR, the use of robotics permits mapping of the interactions of a protein with different interaction partners in a few weeks. As the method can be applied to all protein interactions that can be detected via MAPPIT with a sufficient signal and signal/background ratio, we estimate that up to 35% of the human protein-protein interactions can be analyzed. As MAPPIT is used to validate the interaction pairs obtained from yeast two-hybrid screens [57], [58], [59], [60], [61] and an array-format MAPPIT was designed to screen for interactors of a MAPPIT bait in a prey collection of 10.000 preys [62], an increasing number of protein-protein interactions are validated or detected via MAPPIT. The mode of interaction of these protein-protein interactions can now directly be tested via the random mutagenesis method. For example, we identified more than 100 mutations in the Toll-like receptor 4 (TLR4) adapter protein TIRAP/Mal that disrupt its interaction with TLR4 and/or MyD88 (Bovijn et al., unpublished results). Similarly, the strategy was successfully used to detect mutations in the ring finger protein RNF41 that specifically affect its interaction with new interaction partners (Masschaele et al., unpublished results). In both studies, the effect of the mutations was confirmed via orthogonal interaction assays and activity tests.

To our knowledge, this is the first published strategy that combines random mutagenesis with a broadly applicable method to detect protein-protein interactions in living human cells. Other mutagenesis strategies for identification of interaction interfaces combine random mutagenesis with yeast two-hybrid, ribosome display or phage display [63], [64], [65], [66], [67]. Unlike these methods, MAPPIT takes place in the cytoplasm of an intact living human cell. This can offer an advantage for proteins that require post-translational modifications, such as phosphorylation, for their interaction. Via heteromeric MAPPIT, it is even possible to bring a modifying enzyme into proximity of the bait to promote its interaction with a prey [68]. In heteromeric MAPPIT, the bait protein and its modifying enzyme are coupled to two separate receptor chains with the extracellular part of the α- or β- chain of the GM-CSF receptor. Stimulation with GM-CSF brings the modifying enzyme and the bait in close proximity, while interaction of the modified bait with a MAPPIT prey is detected as in the regular MAPPIT setup.

In our currently presented method, we used degenerated PCR via Mutazyme II or random mutagenesis, as it allows a low mutation frequency necessary to obtain single point mutations [69]. Moreover, it allows all types of transitions and transversions and shows a reasonably balanced distribution of mutations among the different codons [69], [70]. This reduces the risk of an unbalanced distribution of mutations along the sequence, which could bias our analysis.The Mutazyme II allows a good control of the number of mutations by varying the number of PCR cycles and the amount of input DNA. However, the exact conditions probably differ for different proteins and need to be optimized before the MAPPIT analysis.

Many different methods for the introduction of random mutations have been described and most methods are probably compatible with the method presented in this paper. For example, the introduction of mutations via degenerated primers or gene synthesis can allow the introduction of random mutations at specific sites, and increase the number of single amino acid substitutions [64], [71]. Interestingly, scanning mutagenesis methods via mu transposase variants allow the random integration, deletion or replacements of single or multiple codons by one or more specific codons, allowing for example a random replacement of amino acids by a specific amino acid type [72]–[74].

For the development of the random mutagenesis strategy based on MAPPIT, dimerization of Apobec3G and its interaction with Vif and Gag were used as targets. In the absence of a structure of Vif or of the full-length Apobec3G protein, several molecular aspects of the interplay between Apobec3G and Vif remain unclear. In a previous study, the importance of a predicted head-to-head interface in the N-terminal domain was tested via site directed mutagenesis and MAPPIT [10]. The study confirmed the importance of this interface for Apobec3G-Apobec3G interaction, but also demonstrated that the interface is required for Vif binding. The current study shows that the head-to-head interface of the N-terminal domain is also important for binding to Gag. This correlates well with previous studies that showed the importance of the N-terminal Apobec3G domain for the high affinity interaction with Vif and the RNA-mediated interaction with Gag [19], [36], [37]. The C-terminal domain of Apobec3G is the catalytic deaminase domain and is the target of polyubiquitination via Vif [19], [35], [75]. MAPPIT analysis indicates that the C-terminal domain of Apobec3G is also important for interaction with Apobec3G, Vif and Gag. We therefore tried to identify which regions in the N- and C-terminal domain of Apobec3G are involved in the different interactions via random mutagenesis.

A surprising outcome of the random mutagenesis MAPPIT analysis is that no single amino acid substitutions were found that specifically affect only one of the three tested interactions of Apobec3G. A possible explanation for that is that specific binding sites were missed because the coverage of our mutagenesis analysis was too low. However, several arguments argue against this. In the site-directed mutagenesis analysis of the N-terminal head-to-head interface, we found that mutations of 17 residues affected the interaction with Vif, Apobec3G [10] or Gag by more than 50%. The random mutagenesis screen detected 12 of these residues, suggesting 70% coverage. This coverage goes together with some redundancy: 47 of the 114 mutated residues discovered in the screen are found multiple times, often with different substitutions. In the ongoing random mutagenesis analyses of Mal and RNF41, we do find multiple mutations that specifically affect one of the interactions without affecting the other interactions (unpublished results), showing that our strategy is capable of finding specific interaction interfaces.

Out of 149 different single amino acid substitution Apobec3G mutants and an estimated 200 mutants with multiple amino acid substitutions, only one mutant had a very specific effect on the Apobec3G-Vif interaction: a combined A51V/P129S mutation. This suggests that the number of single amino acid substitutions in Apobec3G that can specifically affect the Apobec3G-Vif interaction is possibly limited to a few residues around D128 and P129. In the previous study, we reported that D128A, P129A, T32Q and K99D mutations specifically affect the interaction with Vif [10]. These residues cluster in a small area at the edge of the Apobec3G interface, which may be part of a Vif binding site. Mutating D128 to a lysine, which is the amino acid found at the corresponding position in African green monkeys, disturbs the interaction of human Apobec3G with HIV-1 Vif resulting in resistance to HIV-1 Vif. On the other hand, this Apobec3G D128K mutant is sensitive to African green monkey SIV Vif [76], [77], [78], [79]. The specificity of the effect of a mutation in the D128/P129 area depends on the type of substitution, as a D128G mutation detected in our screen affected the interaction with the three preys, while a site-directed D128K mutation specifically affected the interaction with the Vif prey. This again supports the view that the Apobec3G dimerization site is very close to the Vif binding site. As most mutations that affect Apobec3G dimerization in our MAPPIT assay also affect Vif binding, Vif may bind to an Apobec3G dimer, although we cannot exclude that the Vif binding site simply strongly overlaps with the Apobec3G dimerization site. The interaction of Apobec3G mutants with Gag and Gagpol shows a similar pattern: all mutants that strongly affect the Apobec3G-Apobec3G interaction in MAPPIT affect the Apobec3G-Gagpol interaction.

Apobec3G dimerization requires RNA, as RNAse treatment abolishes the Apobec3G-Apobec3G interaction [10]. In our head-to-head dimer model of the N-terminal domain, many positively charged residues were found in an extended symmetrical surface formed by α helices 2, 3 and 4 of both monomers. In this surface, we found a strong clustering of residues with a high RNA binding propensity, suggesting that this surface is the RNA binding site. This RNA binding site overlaps with, but seems to extend beyond the positively charged pocket in the model of Huthoff et al., which was shown to play a role in association with cellular RNA [51]. Binding of RNA to this putative RNA binding site may be required to overcome the repulsive electrostatic force between the positively charged N-terminal domains. Mutations in the RNA binding site disrupt the interactions of Apobec3G with Apobec3G, Vif and Gag.

Apobec3G forms dimers, tetramers and oligomers. The crystal structure of the C-terminal domain of Apobec3G reveals several intermolecular contacts. The largest interaction interface (901 Å2) corresponds roughly to the head-to-head interface found in Apobec2, although the contacts are quite different. A W211A/R213A/R374E mutation in this interface abolishes the Apobec3G deaminase activity and its antiviral effect [46]. Mutations in this tail-to-tail interface of the C-terminal domain interfere with Apobec3G oligomerization [80]. This leads to a model where Apobec3G forms dimers via the head-to-head interface of the N-terminal domain and further oligomerizes via tail-to-tail interactions between C-terminal domains, as predicted by Wedekind et al. (48). The random mutagenesis screen did not detect disruptive mutants in the predicted tail-to-tail interface of the C-terminal domain. However, it is possible that the MAPPIT assay cannot detect mutations that affect oligomerization of the Apobec3G bait.

A second smaller interface (604 Å2) in the crystal structure of the Apobec3G C-terminal domain contains residues of the β2 strand and the α2 helix. This interface contains two zinc ion binding sites coordinated by H248, H250, C261 and D264. A H248A/H250A mutation does not interfere with Apobec3G oligomerization in vivo [81], and the significance of this interface for the function Apobec3G is unclear. Apobec3G binding to Vif and Gag is affected by mutations in the β2 strand and the α2 helix of the C-terminal domain of Apobec3G. However, H248A and C261A hardly affect these interactions, indicating that the role of the β2 strand and the α2 helix in interactions of Apobec3G is not related to the proposed zinc-mediated Apobec3G oligomerization (Figure S5 in supporting information).

In the crystal structure, Apobec2 dimerizes via direct interactions between the β2 strands. A similar dimerization was predicted between the N-and C-terminal domain of Apobec3G, in a model where the β2 strand of the N-terminal domain interacts with the β2 strand of the C-terminal domain. The discontinuous β2 strand in the different structures of the C-terminal catalytic domain seems to prohibit this mode of dimerization [44], [45], [46], [47], [48]. However, molecular dynamics simulations predict that the β2 strand of the C-terminal domain may be able to adopt a more extended β strand conformation to allow interactions between two β2 strands [51]. Our extensive random mutagenesis analysis does not support dimerization between the β2 strand of the C- and N-terminal domains. Sharply in contrast with the effect of mutations in the β2 strand area of the C-terminal domain, we did not identify any mutations in the β2 strand area of the N-terminal domain that affect Apobec3G dimerization, or its interaction with Gag or Vif. This argues against an interaction between the β2 strand areas of the N- and C-terminal domain.

Our MAPPIT assays are set up to detect clusters of mutations that affect the interaction in this assay, in order to delineate possible interaction interfaces. However, the effect of a mutation in our (and other) interaction assays should not be extrapolated automatically to an effect of the mutation on biological activity. A F268N+K270E mutation in Apobec3G affects the interaction with a Vif prey in MAPPIT and in co-immunoprecipitation. The same mutation had no effect on degradation of Apobec3G by Vif. Different assays may have different sensitivities towards a mutation, as illustrated by several examples of differing results in studies of Apobec3G. Huthoff et al. showed that mutations of Apopbec3G Y124 toW127 do not affect degradation by Vif [55]. Our MAPPIT assay shows a strong effect of these mutations on the Apobec3G-Vif bait-prey interaction, suggesting that the Apobec3G dimer interface may nevertheless play a role in interaction with Vif [10]. In line with this, a more drastic YFW125-127DY mutation in the dimer interface does affect the interaction with Vif in another study [37]. In another example, our MAPPIT data support the finding of Huthoff et al. that W127A and Y124A mutations affect Apobec3G dimerization [51]. In contrast, Khan et al. report that these mutations do not affect Apobec3G-Apobec3G interaction in their assays [82].

In summary, we developed a new strategy to detect random mutations that disrupt a protein-protein interaction in intact human cells. The strategy was able to detect the head-to-head interface for Apobec3G dimerization and demonstrates its role in interaction with Vif and Gag. Moreover, a new area in the C-terminal domain that is important for the interaction of Apobec3G with Vif and Gag was detected. Our MAPPIT analyses thus identified four potential interaction surfaces in Apobec3G: a head-to-head interface, a Vif binding site and an RNA binding site in the N-terminal domain, and a binding site that is involved in binding of Vif and Gag in the C-terminal domain. The random mutagenesis MAPPIT strategy is broadly applicable and offers the advantage that the effect of many mutations of a protein on interaction with multiple interaction partners can be analyzed in parallel. Our approach thus forms a new tool that can help to gain insight in the structure of protein complexes or identify interesting mutants for functional studies.

## Materials and Methods

### Bait, prey and reporter constructs

All constructs used in this report were generated by standard PCR- or restriction based cloning procedures and used primers are listed in table S1 in supporting infomation. Generation of the basic MAPPIT bait receptor plasmid, pCEL, was described elsewhere [9], [83]. This pcDNA5FRT-derived pCEL vector contains the extracellular part of the erythropoietin receptor (EpoR) and the transmembrane and intracellular domain of the leptin receptor (LR) with the tyrosines in the intracellular domain mutated to phenylalanine. Generation of the bait construct pCEL-Apobec3G and the mutant Apobec3G baits represented in table 1 were as previously reported [10]. pCEL-Apobec3G-GFP, used for the random mutagenesis study, contains Apobec3G with a C-terminal eGFP-tag and was obtained in four steps: the NotI-site and stopcodon of pCEL-Apobec3G were replaced by a StuI-site via mutagenesis PCR with the QuickChange® II Site-Directed Mutagenesis Kit (Agilent Technologies) using primer pair 1. This allowed in frame ligation of eGFP to the C-terminus of Apobec3G after StuI-EcoRI-digestion. A BstBI-site was inserted in the Apobec3G sequence using primer pair 2 whereas the SspI-site in the vector was removed with primer pair 3. The resulting construct is further referred to as pCEL-Apobec3G-GFP. pCEL-Apobec3G W127A-GFP was acquired via mutagenesis of pCEL-Apobec3G-GFP with primer pair 4. The pCEL-Apobec3G N-ter construct was generated via the introduction of a stopcodon after the N-terminal domain of Apobec3G in the pCEL-Apobec3G-GFP construct via primer pair 5. This mutagenesis PCR also introduced an extra SacI-site in pCEL-Apobec3G-GFP which allowed the excision of the N-terminal domain to obtain a bait construct with only the C-terminal domain as bait, pCEL-Apobec3G C-ter-GFP. A stopcodon between the Apobec3G domain and GFP was introduced via primer pair 6. Five mutant Apobec3G bait constructs were generated via site directed mutagenesis with the primer pairs 7−11 represented in table S1 in supporting information.

The basic MAPPIT prey construct, pMG2, was generated in the pMET7 vector and contains a part of the gp130 chain in duplicate as previously described [68]. pMG2-Apobec3G, pMG2-VifoptSLQ [10], pMG2-SH2-Bβ [84] and the STAT3 inducible pXP2d2-rPAP1-luciferase reporter [85] were obtained as described before. pMG2-Gagpol was created by inserting Gagpol from pGA-gagpol(160) (Kind gift of Tibotec) in pMG2 after EcoRI-NotI-digestion. pMG2-Gag was obtained via amplification of Gag using primer pair 12 on pNL4-3 (Kind gift of Dr. C. Verhofstede) and ligation in pMG2 after EcoRI-XbaI-digestion. A prey construct with the N-terminal domain of Apobec3G as prey was generated via mutagenesis of pMG2-Apobec3G with primer pair 13 introducing a stopcodon after the N-terminal domain of Apobec3G as well as an extra EcoRI-site. pMG2-Apobec3G-Cter was constructed via EcoRI-digestion of pMG2-Apobec3G N-ter stop and self ligation without the N-terminal domain. pMG2-SVT contains amino acids 261–708 of SV40 large T antigen as prey in the pMG2 vector, and was previously described [68].

### Cell culture and transfection protocol in 6-well and 96-well plates

HEK293T cells (293T/17 obtained from www.atcc.org) were cultured in a 8% CO2 humidified atmosphere at 37°C and grown in Dulbecco's modified Eagle's medium (Invitrogen) with 10% fetal calf serum (Cambrex Corp.). The transfection procedure for 6-well plates was previously described [85]. For 96-well transfections, 10 000 cells were seeded in black 96-well plates. One day later, the cells were transfected with the desired bait and prey plasmid DNA in the presence of the luciferase reporter gene using the calcium phosphate precipitation procedure. 8 wells were transfected with the same bait/prey transfection mix. The day after transfection, 4 of these wells were stimulated overnight with 10 ng/ml hEpo (Roche) or left untreated. Luciferase activity was measured by chemiluminescence in a TopCount luminometer (Canberra Packard) or an EnVision Multilabel Plate Reader (PerkinElmer).

### Western Blot analysis

Expression of the MAPPIT preys was detected using the M2 mouse monoclonal antibody against the FLAG-tag and a fluorescent goat-anti mouse antibody (LI-COR IRDye 800CW) with detection via the Odyssey imager (LI-COR). Expression of the Apobec3G bait was detected using a rabbit anti-Apobec3G antibody (Sigma prestige antibodies) and a goat anti-rabbit peroxidase conjugate (Jackson Immunoresearch) with detection of chemiluminescence (SuperSignal West Pico Chemiluminescent Substrate, Pierce) via autoradiography. V5-tagged MAPPIT baits were detected using a mouse monoclonal anti-V5 tag monoclonal antibody (Invitrogen) and anti-mouse peroxidase conjugate (KPL) as described above.

### Co-immunoprecipitation

HA-tagged Apobec3G was co-expressed with the MAPPIT prey for VifOptLQ, Gag or SVT in HEK293T cells, by Calcium phosphate transfection as described above. Two days after transfection, cells were lysed with 50 mM Tris-HCl pH 7.5, 125 mM NaCl, 5% glycerol, 0.2% NP40 and Complete Protease Inhibitor Cocktail without EDTA (Roche). The lysates were cleared by centrifugation and incubated for 3 hours with 20 µl monoclonal anti-flag M2 agarose beads (Sigma) to precipitate the MAPPIT preys. The beads were washed four times with 1 ml lysis buffer. The precipitated MAPPIT preys and co-immunoprecipitated Apobec3G were released from the beads by incubation with 100 µg/ml synthetic FLAG peptide for 30 min at 37°C. After centrifugation of the beads, co-immunoprecipitated Apobec3G in the supernatant was detected by Western blot using rat anti-HA monoclonal antibody 3F10 (Roche) and fluorescent goat anti-rat antibody (LI-COR IRDye 800CW) with detection via the Odyssey imager (LI-COR).

### Random mutagenesis of the bait

Apobec3G from the pCEL-Apobec3G-GFP bait is randomly mutated using the GeneMorph® II Random Mutagenesis Kit (Agilent Technologies). The N-terminal part of Apobec3G (bp 1−533) was mutated via error prone PCR of pCEL-Apobec3G-GFP with primer pair 14 from which the forward primer contains a SacI- and the reverse primer a SspI-recognition site. The total amount of plasmid DNA added to the reaction was 200 ng and 15 PCR cycles were performed. After digestion with SacI and SspI, the PCR product was introduced in the SacI-SspI opened pCEL-Apobec3G-GFP vector. For the random mutagenesis PCR of the C-terminal part (bp 522−1152), forward primer 15 and reverse primer 15 with respectively a BstBI- and StuI-site, were used. For this reaction, the amount of start DNA was 1000 ng and the number of cycles was 10. The resulting PCR fragments were digested with BstBI and StuI and ligated in the BstBI-StuI opened pCEL-Apobec3G-GFP vector. After transformation of the randomly mutated Apobec3G bait plasmids in E. coli, 1152 different colonies were picked for both mutagenesis reactions and these 2304 colonies were grown overnight in 2xYT medium in 32 96-deepwell blocks. Row A−F of each deepwell block was inoculated with 72 different mutant colonies, row G was inoculated with 12 different wild type pCEL-Apobec3G-GFP colonies, row H was inoculated with 12 different pCEL-Apobec3G W127A-GFP mutant colonies. Automated miniprep of these 32 deepwell blocks was performed using the Nucleospin® Robot-96 plasmid kit and a Freedom EVO 100 platform (Tecan). The DNA concentration was automatically measured using a Magellan UV spectrophotometer (Tecan) and diluted to 6 ng/µl.

### Automated transfection protocol in 384-well plates and MAPPIT analysis

One day before transfection, 3000 cells per well were seeded in black 384-well plates. Cells were transfected overnight with 1.5 ng bait plasmid, 6.5 ng prey plasmid and 4 ng of the pXP2d2-rPAP1-luciferase reporter per well. Automated transfection via the calcium phosphate precipitation procedure was executed by a Freedom EVO 200 platform (Tecan). 8 wells were transfected with the same bait/prey mixture. Thus, one 384-well plate contained bait/prey mixtures of half of a 96-well bait DNA plate (36 mutant Apobec3G baits, 6 wild type control baits and 6 negative control baits) in combination with one prey. The day after transfection, 4 of the 8 wells were stimulated overnight with 10 ng/ml hEpo, the other 4 wells were left unstimulated, so that all bait/prey combinations were assayed in quadruplicate in both unstimulated and ligand stimulated conditions. After another 24 h, luciferase activity in cell lysates was measured using an EnSpire plate reader (PerkinElmer).

### Analysis of the random mutagenesis MAPPIT data

The MAPPIT signal was determined as fold induction of luciferase activity upon Epo stimulation, by dividing the luciferase activity of the 4 Epo stimulated wells by the luciferase activity of the 4 unstimulated wells.The MAPPIT signal of each bait mutant was compared with the MAPPIT signal of the six wild type baits on the same 384-well plate to calculate a normalized MAPPIT signal. The normalized MAPPIT signal is the result of the fold induction of the mutant divided by the median of the fold induction of the six wild types controls on that plate. The 384-well transfections were repeated three times. For each mutant, we calculated a relative MAPPIT signal. The relative MAPPIT signal is the median of the normalized MAPPIT signals of a mutant in three transfection experiments multiplied by 100. The relative MAPPIT signal of a mutant is expressed as percentage of wild type.

Thresholds were determined for the relative MAPPIT signal, as described in the results section. Mutations that gave rise to a relative MAPPIT signal above (SH2-Bβ prey) or below (VifoptSLQ, Gagpol or Apobec3G preys) these thresholds were selected for sequencing of the Apobec3G mutant. Sequences were determined on Applied Biosystems 3730XL DNA Analyzers at the VIB Genetic Service Facility (http://www.vibgeneticservicefacility.be/). The sequences were aligned to the pCEL-Apobec3G-GFP protein sequence using NCBI BLASTx [86]. The resulting protein alignments were concatenated and realigned using MAFFT [87] and visualized with Jalview [88] to identify amino acid substitutions.

### Molecular modeling and visualization

The mutations that affected the relative MAPPIT signal were mapped on the structure of the Apobec3G C-terminal domain [45], or on a homology model of the Apobec3G N-terminal domain [10]. The structures were visualized using UCSF chimera [89] and the mutations were colored according to their relative MAPPIT signal using the ‘render by attribute’ tool of UCSF chimera. To identify possible RNA binding sites, all residues in Apobec3G models were colored according to their statistical potential for RNA binding as defined by Pérez-Cano and Fernández-Recio [90]. The solvent accessibility of residues was calculated via NACCESS (Hubbard,S.J. & Thornton, J.M. (1993), ‘NACCESS’, Computer Program, Department of Biochemistry and Molecular Biology, University College London.”), using a probe radius of 1.4 Å. The relative solvent accessibility (RSA) of residues is calculated as the % accessibility compared to the accessibility of that residue type in an extended ALA-x-ALA tripeptide [91].

## Supporting Information

Figure S1 **MAPPIT principle.** A MAPPIT bait construct is composed a bait protein which is coupled to the C-terminus of a chimeric receptor consisting of the extracellular domain of the erythropoietin receptor (EpoR) and the transmembrane and intracellular part a leptin receptor that lacks STAT3 recruitment sites. In the absence of an interacting prey, the bait is unable to signal via STAT3. The prey protein is fused to a duplication of a fragment of the gp130 receptor chain carrying tyrosine motifs that recruit STAT3 after phosphorylation by JAK2. Interaction between bait and prey in combination with stimulation with Epo thus leads to functional complementation of JAK2-STAT3 signaling and induction of a luciferase reporter.
(TIF)

Figure S2 **A Gag 1–377 prey does not interact with the Apobec3G bait in MAPPIT.** The interactions of an Apobec3G bait with preys for full-length Gag and for Gag 1–377 is determined via MAPPIT. The data are expressed as fold induction of luciferase activity after stimulation with Epo. The SH2Bβ prey and SVT prey are used as a positive and negative control. The MAPPIT signal for the Gag1–377 prey drops to the level of the negative SVT prey control (dotted arrow).
(TIF)

Figure S3 **Expression control of prey and bait proteins.** Western blot analysis of expression of the MAPPIT preys (A) and of selected Apobec3G MAPPIT baits (B), as described in materials and methods. The Apobec3G N-terminal domain is not detected by the anti-Apobec3G antibody, which is directed against the Apobec3G C-terminal domain. The Apobec3G H250A mutant is not expressed, in line with the absence of a MAPPIT signal of this mutant in the SH2-Bβ assay (Figure S5 in supporting information).
(TIF)

Figure S4 **Effect of mutations on the MAPPIT signal with the SH-2Bβ prey.** The luciferase activity before and after Epo stimulation with the SH2-Bβ prey is compared for different baits. This is compared to the MAPPIT interaction of these baits with the Apobec3G prey. A: Luciferase activity after Epo stimulation with the SH2-Bβ prey. B: Luciferase activity without Epo stimulation with the SH2-Bβ prey. C: Fold induction of luciferase activity with the SH2-Bβ prey. D: Fold induction of luciferase activity with the Apobec3G prey. All four baits show a similar luciferase activity with the SH2-Bβ prey after Epo stimulation (A). Only the wild type Apobec3G bait and the Q237K+R238C Apobec3G bait interact with the Apobec3G bait (D). Both baits show a high luciferase activity with the SH2-Bβ prey when not stimulated (B), leading to a lower fold induction of luciferase activity for these two baits with the SH2-Bβ prey (C). In contrast, the F268N+K270E mutant and the negative control bait (receptor without bait) do not interact with Apobec3G (D). These two baits have a low luciferase activity SH2-Bβ prey without Epo stimulation (B) and thus have a high fold induction of luciferase activity after Epo stimulation (C). The capability of an Apobec3G bait to interact with the Apobec3G prey seems to parallel its capability to induce luciferase activity with the SH2-Bβ prey in the absence of Epo stimulation.
(TIF)

Figure S5 **Effect of mutations in a putative zinc-binding motif and of combined mutations in the C-terminal domain.** Interactions between different mutant Apobec3G baits and different MAPPIT preys were determined via MAPPIT. The data are expressed as fold induction of luciferase activity after stimulation with Epo. H248A and C261A mutations have only modest effects on any of the interactions. The H250A mutant Apobec3G bait shows no MAPPIT signal with the SH2-Bβ prey, indicating that the bait is not expressed, which is in line with the Western blot analysis (supporting figure S3). Combined mutations (C261A+F268N, F268N+K270E, Q237K+F268N+K270E, Q237K+R238C+F268N+K270E) in the β2 strand and α2 helix strongly affect the interactions with the Vif, Apobec3G, Gagpol and Gag preys.
(TIF)

Figure S6 **Effect of the F268N+K270E mutation on co-immunoprecipitation of Apobec3G with MAPPIT preys for Gag and VifOptSLQ.** HA-tagged Apobec3G or its F268N+K270E mutant is co-expressed with the MAPPIT preys for Gag and VifOptSLQ in HEK293T cells. After immunoprecipitation of the prey, with anti-FLAG agarose, the co-precipitated HA-tagged Apobec3G is determined via Western Blot. The asterisk indicates the HA-Apobec3G bands, the prey bands are indicated with an arrowhead. A. The F268N+K270E mutant (left panel, lane 2) co-immunoprecipitates less efficiently with the Gag prey than Wild-type HA-Apobec3G (left panel, lane 1). B. The F268N+K270E mutant (left panel, lane 3) co-immunoprecipitates less efficiently with the VifOptSLA prey than Wild-type HA-Apobec3G (left panel, lane 1).
(TIF)

Table S1 **Primers used to obtain the described DNA constructs.**
(DOC)

Table S2 **Random single-residue mutations identified in the N-terminal CDA domain.** Column 1 shows the mutations. The relative solvent accessibility of the mutated residue is shown in column 2. The relative MAPPIT signal (% of WT) of the bait mutants for interaction with the Vif, Apobec3G,Gagpol and SH2-Bβ preys is given in columns 3–6.
(DOC)

Table S3 **Random single-residue mutations identified in the C-terminal CDA domain.** Column 1 shows the mutations. The relative solvent accessibility of the mutated residue is shown in column 2. The relative MAPPIT signal (% of WT) of the bait mutants for interaction with the Vif, Apobec3G,Gagpol and SH2-Bβ preys is given in columns 3–6.
(DOC)
