# Supplementary figures and images for: Random Mutagenesis MAPPIT Analysis Identifies Binding Sites for Vif and Gag in Both Cytidine Deaminase Domains of Apobec3G
Source: PLoS One. 2012 Sep 10;7(9):e44143. doi: 10.1371/journal.pone.0044143 (PMC3438196; doi:10.1371/journal.pone.0044143)

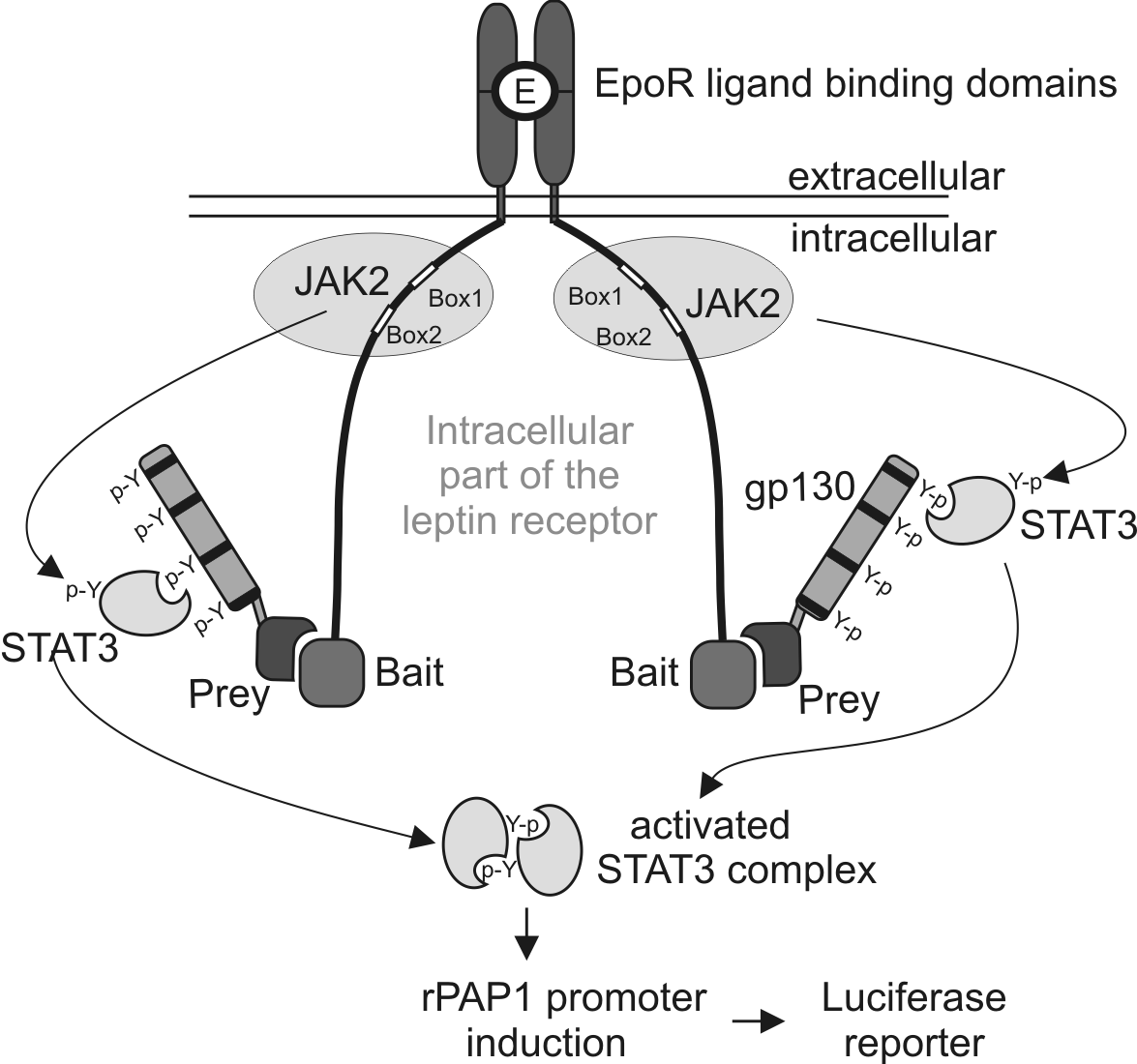

Supplement: Figure S1 — MAPPIT principle. A MAPPIT bait construct is composed a bait protein which is coupled to the C-terminus of a chimeric receptor consisting of the extracellular domain of the erythropoietin receptor (EpoR) and the transmembrane and intracellular part a leptin receptor that lacks STAT3 recruitment sites. In the absence of an interacting prey, the bait is unable to signal via STAT3. The prey protein is fused to a duplication of a fragment of the gp130 receptor chain carrying tyrosine motifs that recruit STAT3 after phosphorylation by JAK2. Interaction between bait and prey in combination with stimulation with Epo thus leads to functional complementation of JAK2-STAT3 signaling and induction of a luciferase reporter. (TIF) [file pone.0044143.s001.tif]

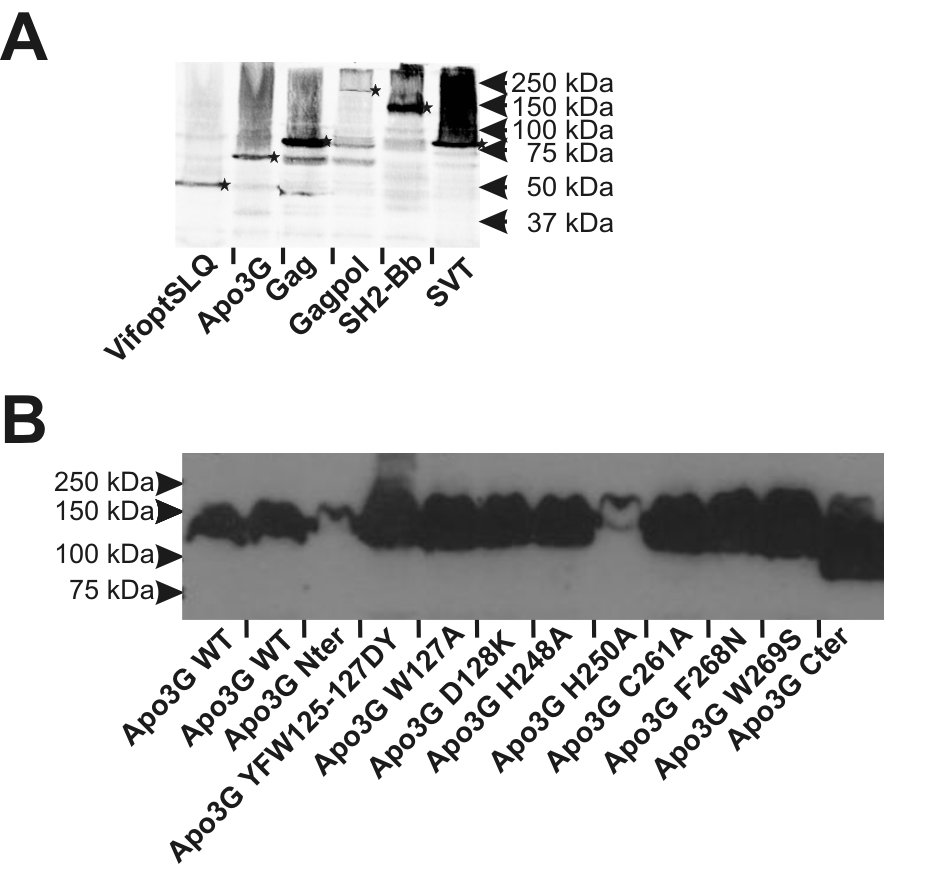

Supplement: Figure S3 — Expression control of prey and bait proteins. Western blot analysis of expression of the MAPPIT preys (A) and of selected Apobec3G MAPPIT baits (B), as described in materials and methods. The Apobec3G N-terminal domain is not detected by the anti-Apobec3G antibody, which is directed against the Apobec3G C-terminal domain. The Apobec3G H250A mutant is not expressed, in line with the absence of a MAPPIT signal of this mutant in the SH2-Bβ assay (Figure S5 in supporting information). (TIF) [file pone.0044143.s003.tif]

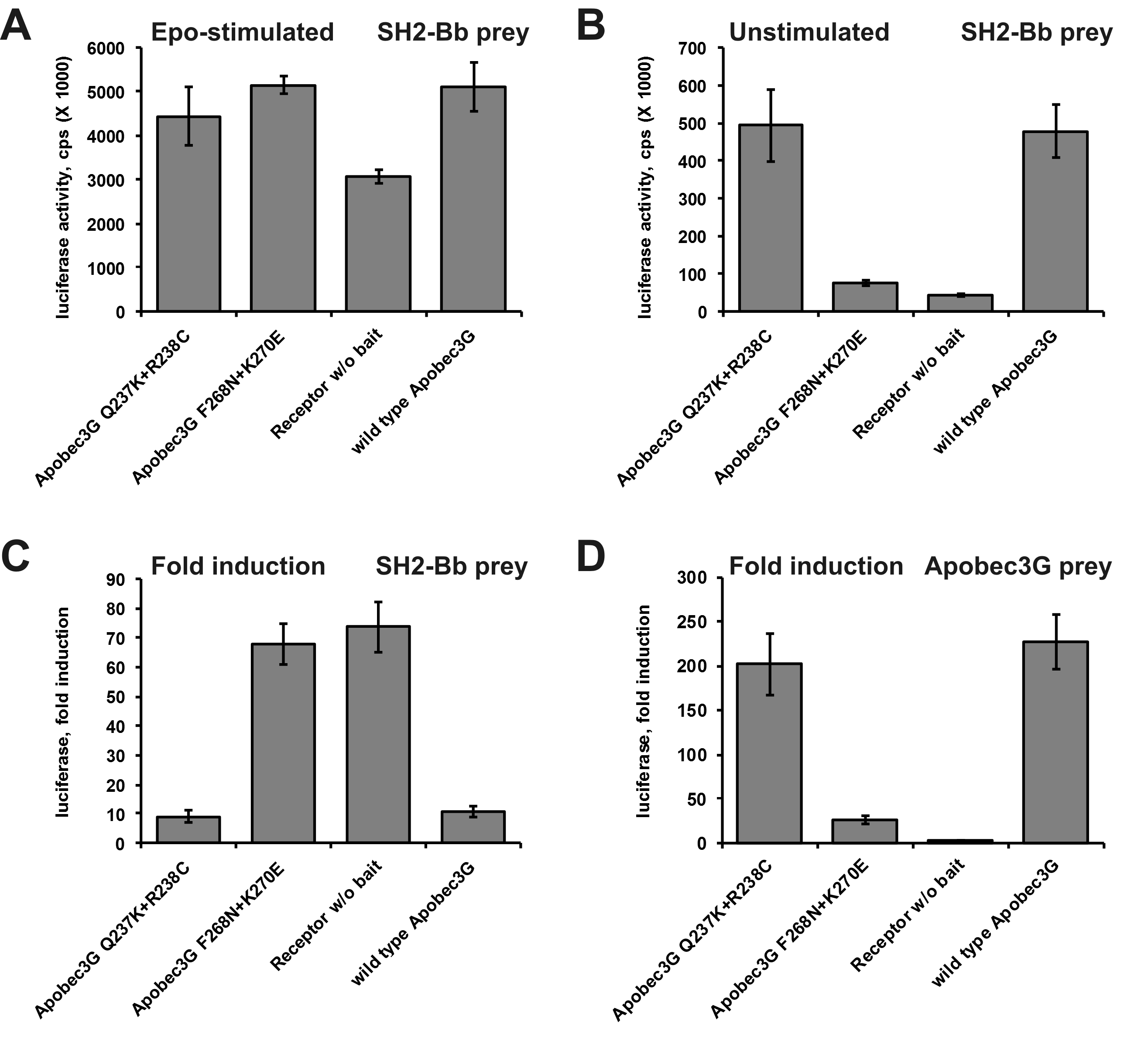

Supplement: Figure S4 — Effect of mutations on the MAPPIT signal with the SH-2Bβ prey. The luciferase activity before and after Epo stimulation with the SH2-Bβ prey is compared for different baits. This is compared to the MAPPIT interaction of these baits with the Apobec3G prey. A: Luciferase activity after Epo stimulation with the SH2-Bβ prey. B: Luciferase activity without Epo stimulation with the SH2-Bβ prey. C: Fold induction of luciferase activity with the SH2-Bβ prey. D: Fold induction of luciferase activity with the Apobec3G prey. All four baits show a similar luciferase activity with the SH2-Bβ prey after Epo stimulation (A). Only the wild type Apobec3G bait and the Q237K+R238C Apobec3G bait interact with the Apobec3G bait (D). Both baits show a high luciferase activity with the SH2-Bβ prey when not stimulated (B), leading to a lower fold induction of luciferase activity for these two baits with the SH2-Bβ prey (C). In contrast, the F268N+K270E mutant and the negative control bait (receptor without bait) do not interact with Apobec3G (D). These two baits have a low luciferase activity SH2-Bβ prey without Epo stimulation (B) and thus have a high fold induction of luciferase activity after Epo stimulation (C). The capability of an Apobec3G bait to interact with the Apobec3G prey seems to parallel its capability to induce luciferase activity with the SH2-Bβ prey in the absence of Epo stimulation. (TIF) [file pone.0044143.s004.tif]

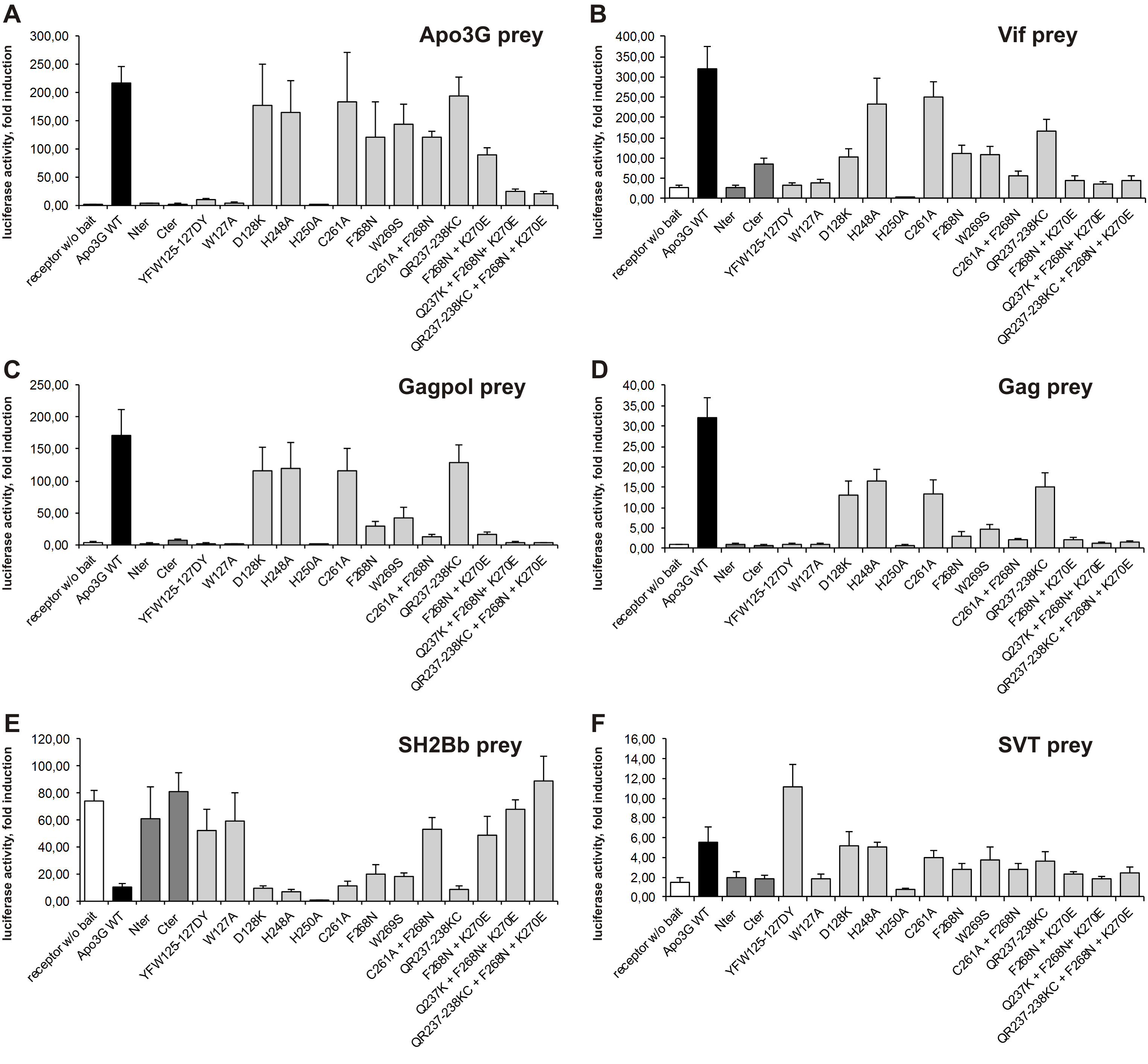

Supplement: Figure S5 — Effect of mutations in a putative zinc-binding motif and of combined mutations in the C-terminal domain. Interactions between different mutant Apobec3G baits and different MAPPIT preys were determined via MAPPIT. The data are expressed as fold induction of luciferase activity after stimulation with Epo. H248A and C261A mutations have only modest effects on any of the interactions. The H250A mutant Apobec3G bait shows no MAPPIT signal with the SH2-Bβ prey, indicating that the bait is not expressed, which is in line with the Western blot analysis (supporting figure S3). Combined mutations (C261A+F268N, F268N+K270E, Q237K+F268N+K270E, Q237K+R238C+F268N+K270E) in the β2 strand and α2 helix strongly affect the interactions with the Vif, Apobec3G, Gagpol and Gag preys. (TIF) [file pone.0044143.s005.tif]

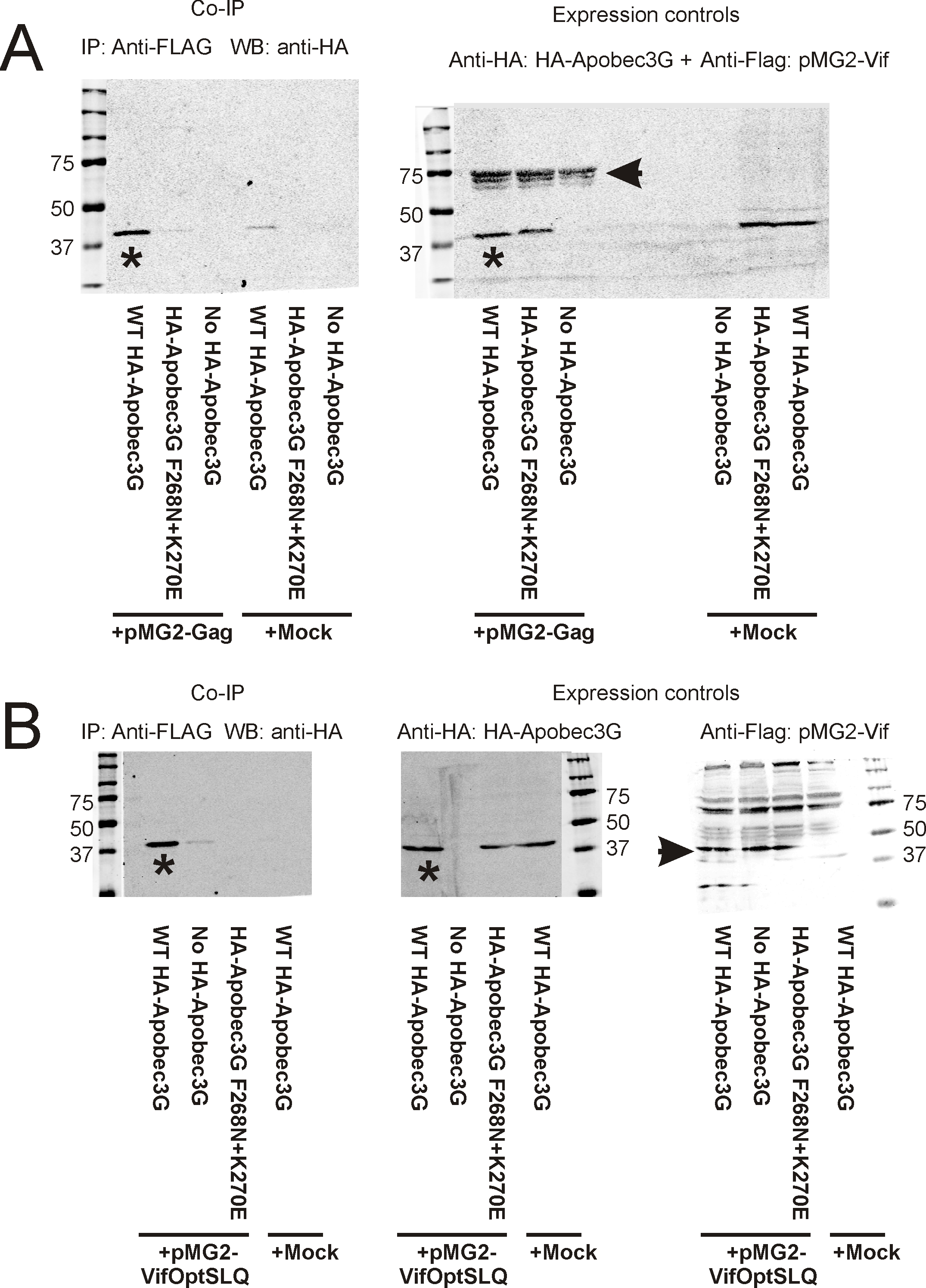

Supplement: Figure S6 — Effect of the F268N+K270E mutation on co-immunoprecipitation of Apobec3G with MAPPIT preys for Gag and VifOptSLQ. HA-tagged Apobec3G or its F268N+K270E mutant is co-expressed with the MAPPIT preys for Gag and VifOptSLQ in HEK293T cells. After immunoprecipitation of the prey, with anti-FLAG agarose, the co-precipitated HA-tagged Apobec3G is determined via Western Blot. The asterisk indicates the HA-Apobec3G bands, the prey bands are indicated with an arrowhead. A. The F268N+K270E mutant (left panel, lane 2) co-immunoprecipitates less efficiently with the Gag prey than Wild-type HA-Apobec3G (left panel, lane 1). B. The F268N+K270E mutant (left panel, lane 3) co-immunoprecipitates less efficiently with the VifOptSLA prey than Wild-type HA-Apobec3G (left panel, lane 1). (TIF) [file pone.0044143.s006.tif]
