# Supplementary material for: Random Mutagenesis MAPPIT Analysis Identifies Binding Sites for Vif and Gag in Both Cytidine Deaminase Domains of Apobec3G
Source: PLoS One. 2012 Sep 10;7(9):e44143. doi: 10.1371/journal.pone.0044143 (PMC3438196; doi:10.1371/journal.pone.0044143)
Supplement: Table S1 — Primers used to obtain the described DNA constructs. (DOC) [file pone.0044143.s007.doc]

|  | **Final construct** | **Forward and reverse primer** |
| --- | --- | --- |
| 1 | pCEL-Apobec3G *-stop-NotI+StuI* | 5’-GAATCAGGAAAACGGAGAGGCCTCTCGAGTCTAGAGGG-3’ 5’-CCCTCTAGACTCGAGAGGCCTCTCCGTTTTCCTGATTC-3’ |
| 2 | pCEL-Apobec3G-GFP *+BstI* | 5’-GCCAAAGAGAGCTATTCGAACCTTGGAATAATCTGCC-3’  5’-GGCAGATTATTCCAAGGTTCGAATAGCTCTCTTTGGC-3’ |
| 3 | pCEL-Apobec3G-GFP *+BstI-StuI* | 5’-CTCTTCCTTTTTCTATCTTATTGAAGCATTTATCAGGG-3’  5’-CCCTGATAAATGCTTCAATAAGATAGAAAAAGGAAGAG-3’ |
| 4 | pCEL-Apobec3G W127A-GFP | 5’-GCCCGCCTCTACTACTTCGCGGACCCAGATTACCAGGAGGCTCTTCGCAGCCTGTGTCAGAAAAG-3’ 5’-CTTTTCTGACACAGGCTGCGAAGAGCCTCCTGGTAATCTGGGTCCGCGAAGTAGTAGAGGCGGGC-3’ |
| 5 | pCEL-Apobec3G Nter *+stop* | 5’-GGGGGAGATTCTCAGACACTGAGCTCGTCGATGGATCCACCCACATTC-3’ 5’-GAATGTGGGTGGATCCATCGACGAGCTCAGTGTCTGAGAATCTCCCCC-3’ |
| 6 | pCEL-Apobec3G Cter *+stop* | 5’-CCATTCTCCAGAATCAGGAAAACTGAGAGGCCTCGAGGGTGAGCAAGGGCGAGG-3’  5’-CCTCGCCCTTGCTCACCCTCGAGGCCTCTCAGTTTTCCTGATTCTGGAGAATGG-3’ |
| 7 | pCEL-Apobec3G H248A | 5’-GGCTTTCTATGCAACCAGGCGCCGGCTAAACACGGTTTCCTTGAAGGCC-3’ 5’-GGCCTTCAAGGAAACCGTGTTTAGCCGGCGCCTGGTTGCATAGAAAGCC-3’ |
| 8 | pCEL-Apobec3G H250A | 5’-CCAGGCTCCACATAAAGCCGGCTTCCTTGAAGGCCGCC-3’ 5’-GGCGGCCTTCAAGGAAGCCGGCTTTATGTGGAGCCTGG-3’ |
| 9 | pCEL-Apobec3G C261A | 5’-GGCCGCCATGCAGAGCTCGCCTTCCTGGACGTGATTCCC-3’ 5’-GGGAATCACGTCCAGGAAGGCGAGCTCTGCATGGCGGCC-3’ |
| 10 | pCEL-Apobec3G F268N | 5’-CTTCCTGGACGTGATTCCCAATTGGAAGCTGGACCTGGAC-3’ 5’-GTCCAGGTCCAGCTTCCAATTGGGAATCACGTCCAGGAAG-3’ |
| 11 | pCEL-Apobec3G W269S | 5’-GGACGTGATTCCCTTTTCGAAGCTGGACCTGGAC-3’ 5’-GTCCAGGTCCAGCTTCGAAAAGGGAATCACGTCC-3’ |
| 12 | Amplification of Gag | 5’-CGTACGAATTCGGGAGCTCGATGGGTGCGAGAGCGTCGGTATTAAGC-3’ 5’-GGTCATCTAGACCGCGGCCGCTTATTGTGACGAGGGGTCGCTG-3 |
| 13 | pMG2-Apobec3G N-ter *+stop* | 5’-GGGGGAGATTCTCAGACACTGAATTCACGATGGATCCACCCACATTC-3’ 5’-GAATGTGGGTGGATCCATCGTGAATTCAGTGTCTGAGAATCTCCCC-3’ |
| 14 | Error prone amplification of N-ter Apo3G | 5’-GGTGGGTCGACGAGCTCCGGATCC-3’ 5’-CCCAGCATGATGTGCAGTAATATATAATATTTAGGC-3 |
| 15 | Error prone amplification of C-ter Apo3G | 5’-CGTGTACAGCCAAAGAGAGCTATTCGAACC-3’ 5’-CCCTTGCTCACCATGGAGGCCTCTCC-3’ |

**Supporting table S1. Primers used to obtain the described DNA constructs.**
