# Supplementary material for: Random Mutagenesis MAPPIT Analysis Identifies Binding Sites for Vif and Gag in Both Cytidine Deaminase Domains of Apobec3G
Source: PLoS One. 2012 Sep 10;7(9):e44143. doi: 10.1371/journal.pone.0044143 (PMC3438196; doi:10.1371/journal.pone.0044143)
Supplement: Table S2 — Random single-residue mutations identified in the N-terminal CDA domain. Column 1 shows the mutations. The relative solvent accessibility of the mutated residue is shown in column 2. The relative MAPPIT signal (% of WT) of the bait mutants for interaction with the Vif, Apobec3G,Gagpol and SH2-Bβ preys is given in columns 3–6. (DOC) [file pone.0044143.s008.doc]

**Supporting table S2. Random single-residue mutations identified in the N-terminal CDA domain.**

|  | **RSA** | **Vif** | **Apobec3G** | **Gagpol** | **SH2-Bβ** |
| --- | --- | --- | --- | --- | --- |
| **F17S** | 17 | 24 | 2 | 5 | 445 |
| **F17I** | 17 | 19 | 2 | 4 | 247 |
| **N20D** | 62 | 51 | 80 | 22 | 256 |
| **F21L** | 8 | 23 | 11 | 4 | 337 |
| **N23I** | 0 | 22 | 2 | 4 | 502 |
| **R24G** | 84 | 32 | 5 | 4 | 360 |
| **L27R** | 68 | 26 | 3 | 3 | 660 |
| **R30L** | 146 | 27 | 13 | 6 | 352 |
| **R30L** | 146 | 21 | 10 | 5 | 186 |
| **L35P** | 0 | 28 | 3 | 4 | 341 |
| **Y37N** | 23 | 25 | 7 | 5 | 301 |
| **Y37N** | 23 | 18 | 7 | 4 | 411 |
| **E38V** | 5 | 27 | 48 | 10 | 358 |
| **E38D** | 5 | 52 | 79 | 29 | 272 |
| **E38D** | 5 | 45 | 69 | 30 | 242 |
| **E38D** | 5 | 43 | 61 | 28 | 261 |
| **E38D** | 5 | 51 | 79 | 25 | 288 |
| **E38V** | 5 | 25 | 50 | 12 | 358 |
| **E38D** | 5 | 46 | 95 | 29 | 295 |
| **V39M** | 0 | 61 | 94 | 45 | 228 |
| **V39K** | 0 | 21 | 2 | 4 | 408 |
| **K40I** | 38 | 59 | 81 | 28 | 197 |
| **G56D** | 0 | 41 | 60 | 17 | 437 |
| **G56D** | 0 | 45 | 62 | 25 | 270 |
| **Y59N** | 103 | 18 | 9 | 3 | 204 |
| **K63R** | 78 | 19 | 5 | 3 | 287 |
| **P66R** | 0 | 20 | 3 | 4 | 339 |
| **W73L** | 165 | 69 | 96 | 44 | 199 |
| **R78W** | 83 | 16 | 18 | 6 | 276 |
| **R78W** | 83 | 34 | 26 | 9 | 328 |
| **K79M** | 112 | 56 | 74 | 32 | 274 |
| **H81L** | 134 | 53 | 70 | 33 | 247 |
| **E87D** | 48 | 16 | 6 | 5 | 281 |
| **W90C** | 0 | 25 | 6 | 5 | 586 |
| **W90G** | 0 | 24 | 3 | 4 | 402 |
| **W90R** | 0 | 22 | 5 | 7 | 283 |
| **S93F** | 5 | 14 | 5 | 3 | 231 |
| **W94R** | 55 | 27 | 14 | 13 | 454 |
| **P96H** | 7 | 21 | 11 | 3 | 330 |
| **P96L** | 7 | 22 | 3 | 4 | 443 |
| **C97S** | 1 | 20 | 6 | 2 | 320 |
| **C97S** | 1 | 39 | 9 | 7 | 327 |
| **C97R** | 1 | 16 | 3 | 4 | 320 |
| **C100S** | 0 | 25 | 5 | 5 | 448 |
| **C100S** | 0 | 30 | 4 | 5 | 533 |
| **C100P** | 0 | 30 | 3 | 4 | 335 |
| **R102G** | 121 | 44 | 74 | 15 | 272 |
| **R102W** | 121 | 83 | 73 | 40 | 202 |
| **L108M** | 2 | 61 | 82 | 52 | 297 |
| **D111G** | 1 | 37 | 54 | 13 | 346 |
| **D111G** | 1 | 41 | 61 | 24 | 311 |
| **D111V** | 1 | 46 | 75 | 22 | 241 |
| **K113M** | 66 | 67 | 96 | 51 | 210 |
| **K113E** | 66 | 40 | 64 | 17 | 257 |
| **V114A** | 6 | 33 | 46 | 10 | 300 |
| **F119L** | 36 | 49 | 61 | 28 | 242 |
| **F119I** | 36 | 38 | 69 | 18 | 364 |
| **F120S** | 0 | 31 | 30 | 8 | 283 |
| **A121V** | 20 | 26 | 30 | 8 | 350 |
| **A121V** | 20 | 26 | 37 | 6 | 360 |
| **A121D** | 20 | 27 | 4 | 5 | 403 |
| **R122C** | 108 | 22 | 3 | 3 | 301 |
| **R122C** | 108 | 16 | 3 | 5 | 339 |
| **L123H** | 2 | 22 | 3 | 3 | 379 |
| **L123P** | 2 | 25 | 4 | 4 | 282 |
| **W127C** | 152 | 18 | 4 | 4 | 360 |
| **W127C** | 152 | 27 | 6 | 5 | 259 |
| **W127R** | 152 | 16 | 4 | 4 | 370 |
| **D128G** | 15 | 19 | 47 | 11 | 356 |
| **D130Y** | 77 | 43 | 72 | 49 | 157 |
| **A134V** | 1 | 56 | 73 | 32 | 223 |
| **L135P** | 9 | 25 | 3 | 4 | 331 |
| **L135H** | 9 | 22 | 7 | 4 | 475 |
| **R136S** | 83 | 53 | 69 | 24 | 243 |
| **L138P** | 10 | 18 | 3 | 4 | 271 |
| **L138Q** | 10 | 18 | 4 | 3 | 426 |
| **C139R** | 2 | 22 | 12 | 5 | 369 |
| **C139S** | 2 | 46 | 65 | 23 | 275 |
| **C139R** | 2 | 18 | 3 | 3 | 397 |
| **C139W** | 2 | 28 | 27 | 5 | 356 |
| **I151T** | 10 | 31 | 40 | 6 | 312 |
| **I151N** | 10 | 18 | 5 | 4 | 346 |
| **M152V** | 3 | 22 | 4 | 4 | 372 |
| **M152V** | 3 | 25 | 4 | 3 | 552 |
| **F157I** | 6 | 39 | 59 | 25 | 341 |
| **C160S** | 0 | 34 | 53 | 9 | 386 |
| **C160S** | 0 | 28 | 56 | 9 | 357 |
| **C160R** | 0 | 36 | 53 | 20 | 421 |
| **W161R** | 10 | 44 | 48 | 19 | 300 |
| **F164L** | 0 | 45 | 21 | 9 | 395 |
| **F164L** | 0 | 31 | 24 | 10 | 382 |
| **F164L** | 0 | 28 | 20 | 6 | 393 |
| **F172I** | 31 | 36 | 50 | 18 | 336 |
| **E173G** | 49 | 51 | 91 | 41 | 198 |
| **W175R** | 50 | 20 | 31 | 8 | 306 |
| **W175R** | 50 | 24 | 28 | 4 | 265 |
| **W175L** | 50 | 60 | 70 | 25 | 344 |
| **N177S** | 59 | 85 | 96 | 45 | 187 |
| **N177Y** | 59 | 101 | 130 | 66 | 133 |
| **L189R** | 2 | 27 | 18 | 9 | 205 |
| **E191V** | 115 | 41 | 40 | 8 | 231 |
| **L193H** | 30 | 20 | 31 | 29 | 164 |

Column 1 shows the mutations. The relative solvent accessibility of the mutated residue is shown in column 2. The relative MAPPIT signal (% of WT) of the bait mutants for interaction with the Vif, Apobec3G,Gagpol and SH2-Bβ preys is given in columns 3-6.
