# Supplementary material for: Random Mutagenesis MAPPIT Analysis Identifies Binding Sites for Vif and Gag in Both Cytidine Deaminase Domains of Apobec3G
Source: PLoS One. 2012 Sep 10;7(9):e44143. doi: 10.1371/journal.pone.0044143 (PMC3438196; doi:10.1371/journal.pone.0044143)
Supplement: Table S3 — Random single-residue mutations identified in the C-terminal CDA domain. Column 1 shows the mutations. The relative solvent accessibility of the mutated residue is shown in column 2. The relative MAPPIT signal (% of WT) of the bait mutants for interaction with the Vif, Apobec3G,Gagpol and SH2-Bβ preys is given in columns 3–6. (DOC) [file pone.0044143.s009.doc]

**Supporting table S3. Random single-residue mutations identified in the C-terminal CDA domain.**

|  | **%RSA** | **Vif** | **Apobec3G** | **Gagpol** | **SH2-Bβ** |
| --- | --- | --- | --- | --- | --- |
| **P199L** | 35 | 80 | 80 | 54 | 205 |
| **N205D** | 8 | 69 | 104 | 29 | 287 |
| **F206I** | 0 | 20 | 42 | 7 | 205 |
| **F206L** | 0 | 39 | 65 | 11 | 360 |
| **N208I** | 2 | 24 | 73 | 15 | 263 |
| **N208Y** | 2 | 34 | 68 | 31 | 339 |
| **N208S** | 2 | 53 | 104 | 39 | 286 |
| **G214R** | 44 | 87 | 89 | 65 | 128 |
| **E217V** | 53 | 87 | 85 | 66 | 105 |
| **L220Q** | 0 | 32 | 69 | 10 | 247 |
| **L220Q** | 0 | 38 | 52 | 18 | 222 |
| **Y222N** | 25 | 12 | 7 | 6 | 116 |
| **E223G** | 18 | 67 | 81 | 31 | 174 |
| **E223G** | 18 | 67 | 81 | 31 | 174 |
| **E223D** | 18 | 60 | 72 | 84 | 117 |
| **E225G** | 23 | 83 | 127 | 66 | 123 |
| **Q237K** | 92 | 80 | 99 | 46 | 251 |
| **R238C** | 80 | 55 | 106 | 56 | 153 |
| **R238C** | 80 | 58 | 87 | 59 | 210 |
| **R238C** | 80 | 72 | 101 | 63 | 133 |
| **R239G** | 45 | 83 | 83 | 48 | 183 |
| **R239G** | 45 | 70 | 82 | 66 | 150 |
| **G240A** | 17 | 66 | 107 | 79 | 200 |
| **F241S** | 68 | 87 | 94 | 62 | 93 |
| **F241L** | 68 | 112 | 113 | 62 | 160 |
| **N244I** | 39 | 43 | 64 | 18 | 204 |
| **N244S** | 39 | 66 | 91 | 111 | 131 |
| **H248Y** | 123 | 98 | 47 | 104 | 141 |
| **E259D** | 8 | 59 | 77 | 29 | 215 |
| **L260P** | 41 | 58 | 95 | 30 | 168 |
| **F262Y** | 0 | 43 | 56 | 19 | 211 |
| **L263Q** | 20 | 30 | 58 | 24 | 287 |
| **D264Y** | 60 | 71 | 47 | 40 | 225 |
| **V265M** | 48 | 92 | 100 | 48 | 198 |
| **I266T** | 8 | 60 | 119 | 47 | 166 |
| **I266F** | 8 | 68 | 132 | 65 | 160 |
| **W269R** | 20 | 43 | 50 | 11 | 190 |
| **W269G** | 20 | 36 | 68 | 33 | 150 |
| **K270E** | 87 | 58 | 54 | 67 | 133 |
| **L271Q** | 24 | 64 | 86 | 24 | 205 |
| **R278G** | 77 | 29 | 54 | 9 | 134 |
| **R278G** | 77 | 37 | 51 | 12 | 239 |
| **F282S** | 8 | 48 | 74 | 18 | 204 |
| **F282S** | 8 | 46 | 85 | 22 | 196 |
| **F282S** | 8 | 38 | 64 | 49 | 337 |
| **S284F** | 0 | 17 | 7 | 4 | 315 |
| **S286R** | 4 | 23 | 52 | 10 | 189 |
| **C291Y** | 1 | 23 | 32 | 7 | 171 |
| **C291S** | 1 | 40 | 73 | 16 | 354 |
| **M295K** | 1 | 39 | 72 | 12 | 228 |
| **A296V** | 8 | 64 | 102 | 85 | 97 |
| **S300P** | 67 | 65 | 97 | 46 | 96 |
| **V305E** | 10 | 17 | 26 | 5 | 151 |
| **F310C** | 57 | 104 | 69 | 130 | 134 |
| **A312T** | 0 | 65 | 80 | 30 | 157 |
| **G324R** | 0 | 42 | 81 | 16 | 250 |
| **G324R** | 0 | 38 | 63 | 23 | 210 |
| **L325Q** | 2 | 31 | 42 | 12 | 252 |
| **L325Q** | 2 | 28 | 44 | 19 | 237 |
| **R326H** | 75 | 57 | 75 | 63 | 129 |
| **L328P** | 0 | 13 | 6 | 3 | 93 |
| **A331T** | 32 | 63 | 90 | 73 | 96 |
| **I337K** | 11 | 59 | 66 | 23 | 126 |
| **I337L** | 11 | 72 | 78 | 62 | 154 |
| **S341C** | 69 | 122 | 111 | 63 | 95 |
| **F343L** | 0 | 101 | 82 | 26 | 199 |
| **F343I** | 0 | 88 | 70 | 49 | 185 |
| **F343L** | 0 | 70 | 108 | 52 | 114 |
| **F358L** | 21 | 71 | 96 | 47 | 182 |
| **P360S** | 71 | 64 | 172 | 136 | 122 |
| **W361R** | 41 | 25 | 77 | 23 | 282 |
| **L364P** | 4 | 31 | 53 | 8 | 245 |
| **D365N** | 76 | 94 | 42 | 94 | 121 |
| **E366D** | 124 | 103 | 100 | 61 | 112 |
| **S368N** | 4 | 46 | 102 | 46 | 264 |
| **S368G** | 4 | 50 | 110 | 59 | 118 |
| **L375Q** | 10 | 37 | 59 | 10 | 281 |
| **L379P** | 59 | 43 | 45 | 15 | 195 |
| **L379P** | 59 | 37 | 69 | 23 | 176 |
| **L379P** | 59 | 35 | 64 | 27 | 129 |
| **Q380H** | 97 | 54 | 117 | 113 | 113 |

Column 1 shows the mutations. The relative solvent accessibility of the mutated residue is shown in column 2. The relative MAPPIT signal (% of WT) of the bait mutants for interaction with the Vif, Apobec3G,Gagpol and SH2-Bβ preys is given in columns 3-6.
